# Intelligent Systems Using Sensors and/or Machine Learning to Mitigate Wildlife–Vehicle Collisions: A Review, Challenges, and New Perspectives

**DOI:** 10.3390/s22072478

**Published:** 2022-03-23

**Authors:** Irene Nandutu, Marcellin Atemkeng, Patrice Okouma

**Affiliations:** Department of Mathematics, Rhodes University, Artillery Rd., Grahamstown 6139, South Africa; irenenanduttu@gmail.com (I.N.); p.okouma@ru.ac.za (P.O.)

**Keywords:** wildlife–vehicle collisions, intelligent systems, sensor, machine learning, human–wildlife, human behavior, animal behavior, machine learning datasets, animal detection systems

## Abstract

Worldwide, the persistent trend of human and animal life losses, as well as damage to properties due to wildlife–vehicle collisions (WVCs) remains a significant source of concerns for a broad range of stakeholders. To mitigate their occurrences and impact, many approaches are being adopted, with varying successes. Because of their increased versatility and increasing efficiency, Artificial Intelligence-based methods have been experiencing a significant level of adoption. The present work extensively reviews the literature on intelligent systems incorporating sensor technologies and/or machine learning methods to mitigate WVCs. Included in our review is an investigation of key factors contributing to human–wildlife conflicts, as well as a discussion of dominant state-of-the-art datasets used in the mitigation of WVCs. Our study combines a systematic review with bibliometric analysis. We find that most animal detection systems (excluding autonomous vehicles) are relying neither on state-of-the-art datasets nor on recent breakthrough machine learning approaches. We, therefore, argue that the use of the latest datasets and machine learning techniques will minimize false detection and improve model performance. In addition, the present work covers a comprehensive list of associated challenges ranging from failure to detect hotspot areas to limitations in training datasets. Future research directions identified include the design and development of algorithms for real-time animal detection systems. The latter provides a rationale for the applicability of our proposed solutions, for which we designed a continuous product development lifecycle to determine their feasibility.

## 1. Introduction

Wildlife–vehicle collisions (WVCs) have progressively increased in both developed and developing economies. Every year, the United States (U.S.) registers 725,000 to 1,500,000 WVC cases, with over 200 humans dying annually. In comparison, Canada records a wildlife roadkill rate of 4 to 8 animals per day [1]. WVCs lead to severe economic losses, with the Ministry of Transportation of Canada spending over 600,000 CAD while cleaning up the collision sites. The U.S. Department of Transportation registered 200,000 WVC cases in 1990 and more than 300,000 in 2004 [2]. Generally, from 1975 to the mid-2000s, there were escalating deaths caused by collisions with cars in the U.S. Excitingly, the death trend has leveled off over the past decade. These deaths increased from 89 in 1975 to 223 in 2007 and declined to 185 in 2019. In 2019, the highest number of WVC fatalities occurred only during July–September [3]. Globally, amphibians and reptiles are the most endangered species, with records showing WVC cases of 180 amphibians and 72 reptiles on tertiary roads from March 2014 to October 2015 in a rural region in eastern Austria [4]. In 2015, the greater Mapungubwe transfrontier conservation area in South Africa recorded 991 and 36 roadkill on paved and unpaved roads, respectively [5]. The latter study shows that unpaved roads recorded less WVCs than paved roads. High numbers of WVCs were both in hot and wet seasons, while cold and dry seasons experienced the lowest numbers. In February 2012, researchers recorded 470 animal mortality rates due to WVCs in just two fortnights in South Africa. Birds had the highest numbers, followed by reptiles, mammals, and amphibians [6]. Roadkill and WVCs also happen in other parts of Africa, such as Tanzania [7,8]. In 2015, John Kioki et al. [7] recorded a death rate of 3% for domestic animals and 97% for wildlife due to WVCs. Their research findings show that roads are a potential threat to wildlife in East Africa.

The WVCs have caused death to humans and animals as well as damage to properties and roads, contributing to environmental conditions [9]. These collisions have affected the safety of humans, property, and wildlife. Despite the escalating numbers of WVCs, several methods adopted help solve the problem of roadkill. These methods include traditional (e.g., fencing, underpasses, and overpasses), intelligent systems (e.g., area cover, buried cable, autonomous vehicles, driver assistants, and break-the-beam), and machine learning algorithms. Some of these intelligent systems do not integrate machine learning algorithms to mitigate WVCs. Nevertheless, these intelligent systems and machine learning algorithms experience false detections when engaged in many accidents with animals on highways. By definition, machine learning is a sub-field of computational intelligence that exploits mathematical tools to make computers function like the human brain. It has gained popularity to address problems that are challenging to tackle by traditional computer science. Machine learning algorithms include but are not limited to support vector machines [10], naive Bayesian [11], and artificial neural networks (ANNs) [12]. The latter algorithm can minimize prediction and detection errors via the gradient descent based on the back-propagation algorithm. This capability of ANNs enables them to easily solve complex problems in different fields. A type of ANN is the feedforward neural network, which features an extraction process that is oriented from the input layers to the output layers, passing through the hidden layers. Moreover, another type of ANN is the recurrent neural network (RNN) [13], long short-term memory (LSTM), and convolutional neural networks (CNN [14]). Long short-term memory can mimic the behavior of randomly generated data over time. The LSTM does this via massive units with various components such as weights, activation functions, and backward and forward neurons connections to handle the vanishing gradient problem, which a very deep CNN would likely fail to address [15].

### 1.1. Problematic

Despite the escalating damage and danger that comes with WVCs, researchers engage in developing engineering solutions to monitor animals and mitigate the WVCs. Traditional approaches are implemented, such as wildlife crossings (e.g., overpass and underpass structures) and the installation of fences together with segments on highways. These traditional approaches are limited and are not always possible with steep rocky slopes and deep snowpack, among others [16]. Because of these limitations, other solutions such as wildlife–vehicle detection systems have received much attention. These include systems such as break-the-beam [17,18,19,20,21], area cover [17,18,22,23,24,25,26,27], buried cable [28,29,30], driver assistance [31], autonomous vehicles [32], and mobile mapping animal detection systems (ADSs) [33,34], as well as roadkill detection [35]. These ADSs are combined with traditional methods to enhance the prediction and detection of animals on the road. They also help alert the drivers to be aware of animal presence and the exact location of animals on the road. Although recent research on ADSs in combination with machine learning has emerged, it is more popular in autonomous cars, followed by driver assistants. In other systems which try to integrate machine learning and ADSs, their research is still in early stages using simulation techniques. The simulation process uses synthetic images with small training and test datasets resulting in low prediction accuracy [19].

Nevertheless, current survey and review studies on mitigating WVCs with machine learning algorithms and/or intelligent systems are non-exhaustive. In 2021, the Said et al. [36] review on ADSs and characteristics of WVCs is vital, and in 2020, Raphaela Pagany [37] discussed WVC influencing factors. However, their works did not focus on machine learning methods that prevent WVCs and current datasets used to mitigate WVCs. Because of these gaps, the motivation for this paper is six-fold:

- Understand and critique the current ADSs used to mitigate WVCs.
- Explore the negative factors contributing to WVCs; these negative factors should act as features when developing intelligent systems that prevent WVCs.
- Discuss and criticize the current systems that integrate machine learning to mitigate WVCs.
- Identify the challenges and the gaps for the reviewed current systems that prevent WVCs and identify potential solutions or new perspectives.
- Propose future research directions.
- Discuss the applicability and feasibility of the proposed solutions.

This investigation will fill the research gaps by comparing these technologies in terms of their strengths, weaknesses, limitations, and the way forward in the field.

### 1.2. Contributions

We analyze negative factors that influence WVCs, such as human behaviors, animal behaviors, road features, and climatic changes. These factors are key features to consider when developing ADSs and machine learning to prevent WVCs. We review existing systems that prevent WVCs, e.g., area cover systems, break-the-beam systems, and other ADSs. We discuss area cover systems classifications with sensor types such as active infrared sensors, passive infrared sensors, passive video sensors, and active microwave radio sensors. Besides classifying with sensor type, we state the strengths and weaknesses of each area cover system. In break-the-beam systems, we use microwave radio, infrared, and laser sensor types. In addition, we discuss the strengths and weaknesses in each study. We also discuss the strength and weaknesses of the buried cable, mobile mapping, driver assistance, autonomous vehicles, and roadkill ADSs. We conduct discussions on different types of machine learning that prevent WVCs. Supervised methods such as support vector machine (SVM) [38], maximum entropy environmental niche modeling [39], CNN [34,38,40], YOLOv3 [41], Xception [34], VGG16 [34], VGG19 [34], ResNet50 [34], inception [34], and unsupervised methods such as K-nearest neighbors (KNN) [19], PCA [38], feature extraction [19], frame differencing [19], template matching [19], LDA [38], LBPH [38], as well as ensemble learning such as random forest [19] have achieved promising results, among others. We find a non-exhaustive work in the literature that explores self-supervised and semi-supervised machine learning algorithms and how they contribute to mitigating WVCs. Equally, we discuss state-of-the-art datasets that could help improve model accuracy and minimize WVCs. Additionally, we record issues and challenges arising from the review and suggest a way forward. Furthermore, we explain the applicability and feasibility of the proposed solutions to existing damaged roads, accident car collisions, humans, animals, and the environment. Finally, we design a continuous product development lifecycle to help engineering teams determine the feasibility of the proposed solutions.

### 1.3. Manuscript Organization

The organization of this work is as follows: Section 2 discusses the methodology adopted in this work. Section 3 discusses four factors contributing to human–wildlife conflict and eventually WVCs. Section 4 reviews existing intelligent systems that prevent WVCs. These are area cover systems, break-the-beam systems, driver assistants, autonomous vehicles, roadkill detection, mobile mapping systems, as well as buried cable ADSs. Section 5 shows a collection of the datasets used to mitigate WVCs. Section 6 conducts a survey of machine learning methods and how they prevent WVCs. Section 7 identifies issues from the review and proposes potential future research directions. The same section also discuss the applicability and feasibility of the proposed solutions.

## 2. Methodology

To define the methodology supporting this review, we recall at least three dominant approaches in literature reviews: the narrative review, the systematic literature review, and the bibliometric analysis.

The narrative literature review typically provides a broad overview of a specific topic chosen by the author. It is based on the literature available about the designed topic. It is often descriptive and written in an easily readable format [42,43]. The systematic literature review has two objectives: first, perform an extensive literature search through a very detailed process and then critically assess the selected literature [42,43]. Last but not least, the bibliometric analysis aims at developing a quantitative investigation and relies on statistical methods to evaluate several characteristics of specific bibliographic information such as journals, research institutions, geographic location, and other quantifiable characteristics [44].

In the present work, we conduct a combination of a systematic review with some elements of bibliometric analysis.

### 2.1. Formulating Review Questions

The study is guided by formulating a comprehensive list of research questions. The questions guided reviewers on the focus, scope, applicability, and feasibility of the research. We define the study’s goal to draft the review questions easily. We aim at reviewing the literature on intelligence systems using sensor technology and/or machine learning to mitigate WVCs. We focus on reviewing datasets that help mitigate WVCs. Additionally, we define a list of issues and challenges from the review and explain the applicability and feasibility of the proposed solutions. To achieve the aim, the authors formulate and implement research questions in Table 1 to guide them in writing this manuscript. The questions help the authors define the scope of the review and set out what they wanted to answer.

### 2.2. Search Databases

The search databases for this study are Scopus and Google Scholar. The articles are peer-reviewed (PR) and non-peer-reviewed (NPR). We further search specific websites that had relevant information. Scopus is an international database of PR publications from all over the world [45]. Using Google Scholar, an advanced search engine, helps to cover citations that are not in Scopus [46]. We search each database and analyze results based on the search string.

### 2.3. Locating Studies through Searching

To identify relevant publications, we perform a bibliographic search both in Scopus and Google Scholar during April 2020 and November 2021. We also extract content from specific websites which had reliable information. We extract and review a total of 136 manuscripts in this study. To extract manuscripts to review, we use search terms and words such as “animal-detection systems”, “break-the-beam”, “area-cover”, “mobile-mapping”, “buried cable”, “driver assistance”, “unmanned aerial vehicles (UAVs)”, “drone”. To specifically retrieve only manuscripts associated to ADSs that help mitigate WVCs, and those that publish datasets to help reduce WVCs, the term “datasets + wildlife-vehicle collisions” is considered. Detailed general terms and terms for each instance are shown in Table 2. Further, the words “machine learning methods + mitigation + wildlife-vehicle collisions” helped retrieve only manuscripts that use machine learning methods to mitigate WVCs.

### 2.4. Inclusion and Exclusion

**Figure 1:**
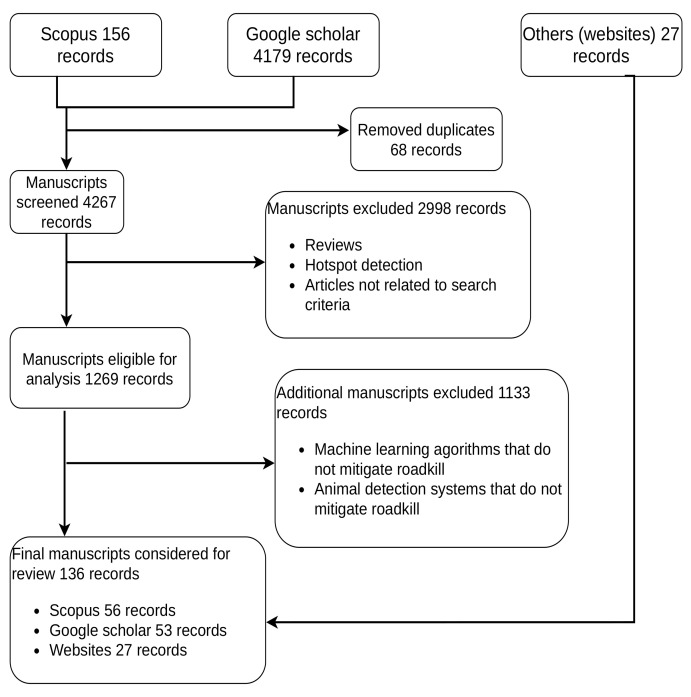
Flow diagram of exclusion and inclusion process.

Our methodology identifies eligible articles we obtain from Scopus and Google Scholar datasets. We compare manuscripts and remove duplicates. Later, we screen the remaining manuscripts for inclusion and exclusion based on defined criteria; this process is shown in Figure 1. We also obtain additional information from websites. Information from websites and Google Scholar also contain company reports.

For any manuscript included in this review, we consider studies that:

- Refer to the use of ADSs and machine learning methods that mitigate WVCs.
- We discuss datasets used to mitigate WVCs.

We exclude studies that:

- Focus on ADSs or machine learning that count and monitor species minus mitigating WVCs.
- We did not consider any machine learning algorithms that detect hotspots.
- Articles that did not satisfy the search criteria.
- Review papers.

### 2.5. Data Extraction and Analysis

**Figure 2:**
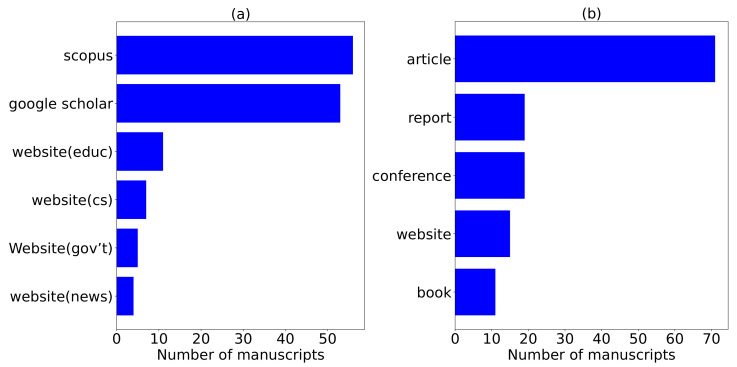
(**a**) Displays the total number of manuscripts extracted from each internet source, and (**b**) visualizes the total number of manuscripts associated to each document type.

**Figure 3:**
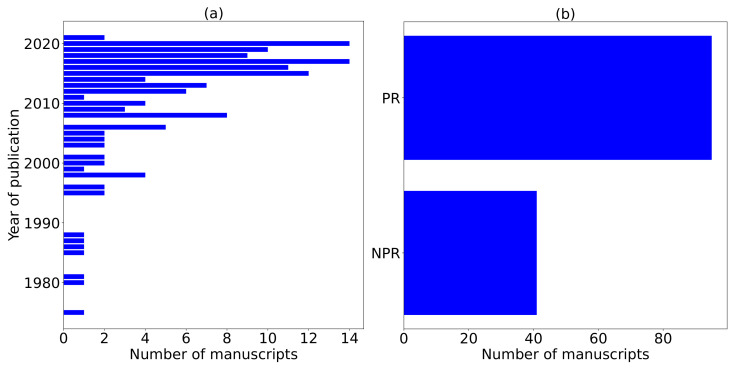
(**a**) Counts manuscripts published per year, and (**b**) summarizes review status of manuscripts, i.e., NPR and PR manuscripts, respectively.

**Figure 4:**
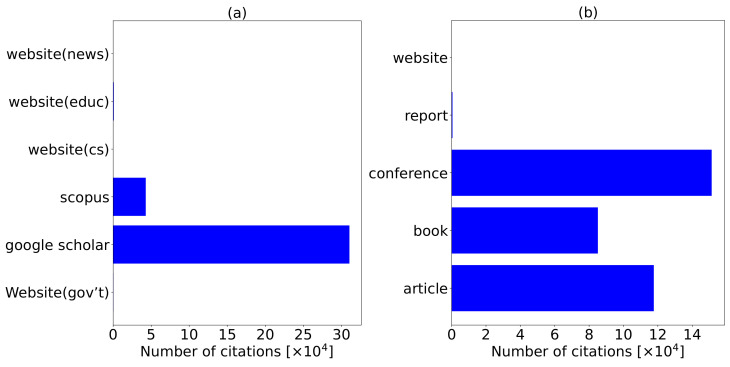
(**a**) Displays the total number of citations per database, and (**b**) visualizes the number of manuscript citations per document type.

**Figure 5:**
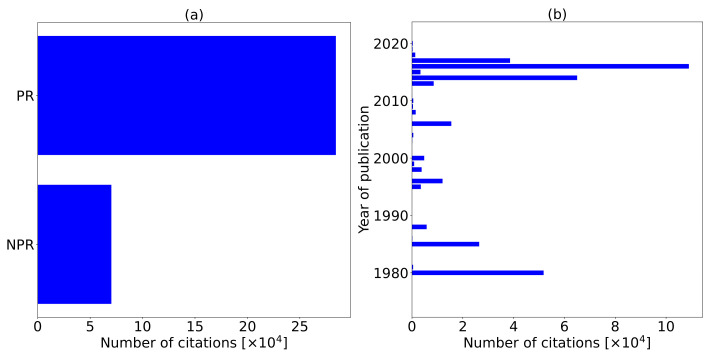
(**a**) Visualizes manuscript citations per review status showing NPR and PR manuscripts, and (**b**) shows relationships between the number of citations per each manuscript and year of publication.

We consider a total of 56 and 53 manuscripts from Scopus and Google Scholar, respectively. Additionally, the authors collect manuscripts from government websites (gov’t), conservation websites (cs), education websites (educ), and news websites (news) with a total of 5, 7, 11, and 4 manuscripts, respectively, totaling to 27 additional manuscripts. Although we find these manuscripts suitable for review, they did not appear in both Scopus and Google Scholar databases. The 27 manuscripts have clear objectives and accurate information which prompted the authors to consider them for review. We summarize and analyze these manuscripts in Figure 2, Figure 3, Figure 4 and Figure 5.

Figure 2a visualizes the total number of manuscripts per each internet source. We extract the highest number of manuscripts from Scopus followed by Google Scholar, education websites (website (educ)), conversational websites (website (cs)), government websites (website (gov’t)), and finally from news websites (website (news)). Figure 2b visualizes the different document types reviewed with the associated number of manuscripts. The latter figure ranks articles as the highly reviewed manuscripts in this review followed by reports, conference papers, then websites and books. It is worthy to note that these document types contain both PR and NPR manuscripts.

Figure 3a shows the distribution of years captured in this review. For each year, we show the total number of manuscripts. The coverage of the manuscripts in this review range from 1975 to 2021, with some years seen as inactive. We observe that research in this field has been neglected, with fluctuations in the yearly number of papers published from 1998 to 2021. The highest number of papers published per year are 14 manuscripts. This shows that more research is required in this field in mitigating WVCs using intelligent systems and machine learning methods. Even though the number of manuscripts progressively published are few, the authors majorly archive them in PR journals as shown in Figure 3b. Figure 3b shows PR and NPR manuscripts. PR manuscripts are many compared to NPR manuscripts. Both PR and NPR manuscripts may contain articles, reports, conference papers, and books; document types are presented in Figure 3b.

To evaluate the relevancy of the research conducted and databases considered, we extract the number of citations as shown in Figure 4. In Figure 4a, we visualize the number of citations per each manuscript associated to a specific database. We observe that manuscripts we extract from the Google Scholar database are cited highly compared to the Scopus database. Google Scholar, Scopus, website (educ), website (gov’t), website (cs), website (news) have 310,593, 42,880, 839, 299, 2, and 0 citations, respectively. The content from websites is composed of company reports. Google Scholar is cited more than the Scopus database due to the following influencing factors:

- Scopus contains only PR manuscripts from Scopus indexed journals.
- Google Scholar contains both PR and NPR manuscripts such as technical which we consider in this review.
- Scopus citation count includes only the number of times the publication was cited by articles from journals that Scopus covers.
- Google Scholar counts citations from every journal published; it may include document types such as books, conference proceedings, dissertations/thesis, patents, technical reports, or other types of publications.

The influencing factors justify the high number of citations witnessed for manuscripts extracted from the Google Scholar database. Based on this, we find the Scopus database very relevant. Figure 4b shows document types with associated number of citations. The number of citations from each document type show that conference papers are highly cited with 151,318 citations followed by journal articles with 117,646 citations, books with 85,106 citations, reports with 516 citations, and 21 citations recorded for websites; citations from websites are possible because websites contain company reports that we include in this study.

Using the same principle, Figure 5 also shows the relevancy of the manuscripts when correlated to the number of citations. Figure 5a visualizes the number of citations of each PR and NPR manuscript. The PR manuscripts are highly cited compared to NPR manuscripts. Figure 5 shows the number of manuscripts per each total number of papers per year. In 2016, the published manuscripts are highly cited, followed by 2014, 1980, 2017, 1985, among others. NPR manuscripts equally have many citations showing that the research published is vital to the domain. The NPR manuscripts have 70,251 citations, while PR manuscripts have 284,362 citations.

Due to the non-exhaustive research conducted on ADSs and machine learning methods that mitigate WVCs, we consider a broad scope from 1975 to 2021 as displayed in Figure 3a. Figure 3a justifies that there is no exhaustive systematic research conducted in this area since there are fluctuations in publications in the different years, with many inactive years showing no research published in the domain. The number of citations in Figure 5b for each manuscript published each year equally fluctuates, with 2016 having the highest number of citations. Due to these unidentified research publication trends, researchers should engage in publishing research that mitigates WVCs.

## 3. RQN1: What Are the Negative Factors That Lead to the Occurrence of WVCs?

Globally, wildlife accidents with cars pose a safety threat to both animals and humans. In this section, we explore the factors influencing the occurrence of these human–wildlife accidents plus attacks. The influencing factors include human behaviors, animal behaviors, road features, and changes in climatic conditions. These factors warrant a detailed description and understanding as they are crucial features to consider during the implementation of intelligent systems that aim to prevent WVCs.

### 3.1. Road Features

A road is a path over which vehicles and other traffic may lawfully pass. It includes pathways and may also include culverts, bridges, and land required for future widening [47]. Roads are characterized by curve road sections, no road marking, smooth, rut, corrugations, express roads, primary roads, secondary roads, double lane line marking, and rural location [48]. In road engineering and construction, the classification of a good road is by its shape, width, thickness, and the materials it is made of [49]. Materials of the road are usually found on a railway crossing, cobbled road, pothole, damaged road, speed bump, manhole covers, and a road in good condition [50]. Roads are a barrier to animal movement as they contribute to roadkill during crossing attempts or behavioral avoidance. WVCs have negative consequences, such as demographic and genetic, that can ultimately result in local or regional extinction [51]. Possibility of extinction is minimized by implementing road structures such as fences to avoid animals from moving toward the roads [52]. The fences limit the movement of animals and therefore reduce roadkill and mortality rates. However, some animals can still jump through these constructed fences and attempt to cross the roads. Road sections with high rates of WVCs are associated with areas with high habitat diversity, limited crop cover, and fewer number of structures [53]. Research shows that there is a significant decline in WVCs when road features such as fencing and pavement are adopted [54]. Collinson et al. [55] designed a low-level roadside fence to direct animals to underpasses as a way of mitigating WVCs of small vertebrates. The study was not satisfactory due to low witnesses of dead animals detected after the installed barrier sites. This study justifies that traditional structures do not totally eliminate WVCs, and the domain requires more research to identify traditional structures or intelligent systems that can mitigate WVCs with extremely high accuracy.

In another work, records of WVC hotspots are more on highways for mammals and birds and riverside roads for amphibians and reptiles [56]. WVCs are more likely to occur alongside roads with thick vegetation cover or habitats which hinder driver visibility [7]. In 2013, Seiler [57] emphasized that the mortality of these animals increases due to high traffic speeds and volume. Road traffic accidents kill hundreds to millions of animals every year, posing a critical threat to the populations of many species [58]. World Health Organization (WHO) states that over 90% of the world road fatalities occur in low-income and middle-income countries, even though these countries have only about half the world’s vehicles. Driver injuries increase due to factors such as lateral crosstown roads, less traffic volume, increase in the percentage of heavy vehicles, wider lanes, lack of road markings, and violation of driving laws and regulations [59]. Road safety is one of the considerations in support of the 2030 agenda for sustainable development [60]. As a mode of contributing to sustainable development goals, the United Nations conference on trade and development wrote a report on road safety, contributing toward the WHO road safety agendas. The document records show that more than 3500 road deaths occur worldwide every day and 1.25 million each year, with the addition of up to 50 million persons facing injuries in road accidents [61]. Furthermore, the annual road traffic predicts that deaths are to increase to around 1.9 million by 2030 and become the seventh leading cause of death [62]. The draft report on road safety states that increase in mortality rates is due to overspeeding, drinking and driving, and lack of motorcycle helmets and seat belts. To address this problem of WVCs, some types of road mitigation measures are available that aim to reduce wildlife mortality on roads. Some of these measures are traditional methods, e.g., overpasses, fences, and underpasses that prevent animals from accessing roads—these traditional solutions minimize the death rate of wildlife. For an effective and reliable approach to avoid WVCs, we propose adopting traditional methods together with ADSs and further integrate them with machine learning algorithms as a mitigation strategy since it contributes to reducing mortality rates [52]. In this review, we give a comprehensive list of proposed solutions for road safety in Section 7.2.

### 3.2. Climate Change and Season

Climate change inconsistencies are a threat globally and are now slowly affecting Africa [63,64]. These changes in seasons are reactions of the greenhouse effect by greenhouse gases. Greenhouse gases include carbon dioxide, methane, dinitrogen monoxide or nitrous oxide, water, perfluorocarbons, and sulfur hexafluoride. As well, halocarbon gases are composed of trichlorofluorocarbon and dichloroflurocarbon. These atmospheric concentrations continue to be possible due to anthropogenic activities (human activities) that hinder the balance of atmospheric gases [65]. The climatic change also creates different weather variations and seasons such as dry (drought) and wet (food) conditions, number of hurricanes, and temperatures [66]. These weather alterations affect bird life and animal life with their behaviors, foraging, migration, growth, and reproduction patterns. When the weather variations affect bird and animal lives, bird and animal movement patterns fluctuate, leading to variations of WVCs on highways. Besides, global warming contributes to a shift in seasons. The variations in seasons contribute to species extinctions since many of the species live in areas that are severely affected by climate change, and climate change is happening too quickly for many species to adapt [67]. Lately, global warming is intriguing. In addition, it has led to the global decline in wildlife, leading to wildlife effects such as the loss of habitats, damage to oceans, coastlines, and coral reefs due to sea-level rise, and increased storm activity, as well as more frequent and intense periods of drought [68].

Climate change and seasons alter wildlife movement and distributions due to the change in position and location of habitats, potentially leading to associated location changes in WVCs. The proposed development of movable structures can be a solution other than overpasses and underpasses. Besides, it is transferable from one location to adapt to the changing weather conditions that affect the animals [69]. In 2012, the Food and Agriculture Organization of the United Nations stated the consequences of climate change. The adverse effects are: (1) The changes in ecosystem and landscape, which reduces biodiversity, leading to significant changes in disturbance patterns, e.g., more fires, more droughts, and more floods, as well as loss of species due to mistiming, competition, and stress from new species within an ecosystem. (2) The variations in species distribution due to temperature, rainfall, and geographical barriers. It is important to note that natural and human-made obstacles to movement are a problem to many animals as they try to move. These animals move to search for new conducive habitats in response to changing conditions. (3) The human–wildlife–livestock conflicts due to increasing human population densities and encroachment of human settlements and activities into wildlife habitats contribute to rampant conflicts between humans and animals. These are majorly common in rural areas where humans depend more on agriculture for farming. These conflicts pressure animals since their habitats have been encroached on. (4) Wildland fires caused by climate change. These affect slow-moving animals at risk of mortality from flames and smoke. Other animals change their habitats due to lack of food, competition for the same territories, or accessing shelter and death due to starvation, and (5) wildlife health, diseases, invasive species, and pests.

The impact of climate change is rampant and includes permanent changes in physical conditions. Examples of these changes are snow cover, permafrost, sea level, and extreme increases in the irregularity and severity of extreme weather events such as droughts, floods, and storms [70]. The impact forces animals to loiter away from their habitats in search of a conducive environment such as green pastures. This act leads them to highways or crossroads. Animals found on the highways usually collide with vehicles leading to WVCs, which increase rampantly. In 2017, in Brazil, the study evaluates variables such as seasonality, climatic (mean temperature, relative air humidity, and accumulated precipitation), and average daily traffic on vertebrate roadkill rates [56]. The highest numbers of roadkill records in the rainy season are reptiles. Roadkill was high for birds during summer, mammals increase from spring to fall, and during fall, the roadkill for amphibians and reptiles was at its peak [71].

### 3.3. Human Behaviors

The behaviors of humans are a threat to conservation in wild reserves. As such, contributing to habitat loss through deforestation, as well as poaching, wildfires, conurbation, and climatic changes in the earth’s atmosphere. The climatic changes are a result of the released amounts of greenhouse gases and aerosols (small particles), burning fossil fuels that release carbon dioxide into the atmosphere. Because of this damage to the atmosphere that humans cause through anthropogenic activities, most blame for climate and season alterations is partially due to their behaviors. Humans possess different behaviors from person to person. Each human has different abilities, aspirations, motives, perceptions, values, attitudes, and personality. Concepts such as actions, activities, and behaviors are essential in understanding human behavior [5]. Actions are short and visible movements such as taking a key, opening the door of a vehicle, and grasping. These actions can also be invisible such as perceptions, attitudes, thoughts, feelings, and physiological processes. On the other hand, activities are composed of several actions such as cutting down trees, poaching, and driving a car. When we combine activities and perform them at different time intervals, we refer to them as behaviors. Behaviors are those such as overspeeding, frequent braking, and poaching, among others [9]. It is worthy to note that actions and activities all contribute to WVCs on highways.

The mentioned activities and actions are driven by the underlying cognitive, emotional, and physiological processes [72]. Social psychologists believe that attitudes contribute to human behavior theory, e.g., what people think and do. They rely majorly on the attitude-based questionnaire by Ajzen and Fishbein [73]. This attitude-based questionnaire has four components that eliminate the difficulty in recognizing the attitude–behavior relationship. These components are beliefs, attitudes, intentions, and behaviors.

Research contains majorly two cognitive theories, which are the theory of reasoned action (TRA) [73,74] and its extension, the theory of planned behavior (TPB) [75]. These theories offer conceptual frameworks for understanding human behavior. In TRA theory, behavior intention combines a person’s attitudes and subjective norms. TPB theory is an extension of TRA theory. TPB states that behavior intentions are usually determined by a combination of a person’s attitudes, subjective norms, and perceived behavioral control. The TRA theory states that a person’s intention to perform or not to perform a behavior at a specific time and place, as well as the more the person thinks other people (friends, family, or society) would love them to perform that behavior (subjective norms), are the immediate determinant of that action, i.e., intentions (behavior intentions) to perform the behavior. This TRA theory is composed to help predict and explain volitional behavior. TRA components are the determinants of intentions, such as attitudes toward behavior determined by beliefs about the behavior and subjective norms. Psychologists define attitude as a tendency of an individual to evaluate an entity such as a person, place, behavior, or thing. The evaluation performed should be with a degree of favor or disfavor [76]. Attitudes are composed of a combination of behavioral beliefs (strong beliefs regarding behavioral outcome) and outcome evaluation (evaluation of the advantages and disadvantages of behavior outcome) [77]. Psychologists emphasize that attitude toward an object is not related to the attitude to behavior toward that object. For example, someone may have a favorable attitude toward a rhino (object). However, they will go ahead and engage in poaching (behavior toward objects) simply because they want the rhino horn, believed to be medicinal. A subjective norm is what we think other people will think of us if we perform (or do not perform) the behavior. Subjective norms are composed of normative beliefs (strong beliefs regarding how important people will agree or disagree with the behavior) and motivation to comply (motivation to comply with important people) [77]. Subjective norms are a result of social and environmental surroundings. Social norms and taboos play a significant role in determining appropriate behavior in society and the motivation to adopt the behavior. In 1988, while conducting a meta-analysis, Sheppard et al. [78] showed that TRA is a powerful predictor of behavior. In general, Ajzen et al. [74] highlight that the more positive a person regards attitude and subjective norm as important, the more likely they are to form intentions to engage in the behavior.

The TPB theory focuses on perceived behavioral control; it looks at a person’s belief in self-control over their behavior. TPB’s key component is perceived behavior control. Perceived behavioral control refers to a person’s perception of how easy or difficult it is to perform any behavior of interest to them. Perceived behavioral control varies across situations and actions, which results in a person having varying perceptions of behavioral control depending on the situation. Perceived behavior control is composed of control beliefs and power beliefs [77]. Control beliefs are strong beliefs about what allows or avoids the performance of the behavior. Power beliefs are power perceived presence of factors that may limit or enhance the performance of a behavior. Human decision-making traits are an attitude of a person toward the behavior, subjective norms regarding behaviors, and a person’s perceived control over the behavior [79]. Generally, a positive attitude and positive subjective norms result in greater perceived control. These increase the likelihood of intentions governing changes in behavior. In other social research, there is an alternative approach that predicts human behavior. The approach looks at behavior aspects of the lives of people as the basis of the question design [80]. This new approach emphasizes replacing attitude questions with questions about the respondent’s environment, consciousness, knowledge, and behavior [81]. Drivers and other stakeholders collide with vehicles due to the power that lies within the two models, TRA and TPB. Their actions and activities are highly attributed to human behaviors. In Table 3, the strengths and limitations of the TRA and TPB models are highlighted.

### 3.4. Animal Behavior

Animal behavior refers to ways in which animals associate with other organisms and the physical environment [72]. On the other hand, behavior is the change in the activity of an animal in response to an external or internal cue [83]. The field of animal behavior is composed of research on: (1) feeding behavior [84], (2) habitat selection [85], (3) mating behavior [86], and (4) social organizations [87]. Animals classified according to their feeding behavior are, e.g., herbivores, carnivores, omnivores, scavengers, and vegetarians. In 1986, behavior evolution was based on assumptions that specific genes hardwire innate behaviors. As well, learned behavior is attained by a more adaptive nervous system than innate behavior. As such, the ability to learn is phylogenetically more recent than innate behavior [88]. These assumptions lead to two categories of behaviors which are innate while others are learned [89]. The categories are determinants of how animals behave the way they do. The physiology and anatomy of an animal also contribute to behavior—both external stimuli (threats from other animals, sounds, smells, weather) and internal stimuli (hunger, fear) prompt behaviors [90]. Both innate and learned behaviors lead to WVCs as animals make decisions to frequently cross the roads.

In wildlife conservation, harsh living conditions, e.g., drought, destabilize animals tempting them to move toward roads [91]. Animal movements are disadvantageous and lead to a lot of negative results, e.g., (1) a decline in biodiversity (due to loss of habitats), (2) the introduction of exotic species (foreign, non-indigenous, or non-native species), (3) over-harvesting of biodiversity resources (illegal hunting, cutting down trees), as well as homogenization of species in agriculture (species lose their “character” through particle size reduction in cells in animals or plants) [92]. WVCs lead to a decline in diversity of animal species in wild reserves. The movement patterns of different animal species toward particular roads continue to expose many repertoires of movement patterns that vary in duration and complexity [93] due to animal behaviors [94].

To enable conservationists and researchers to understand animal behavior easily, implementation of animal tracking technology in game reserves has been ongoing and helps to capture and extract behavioral data [95], using monitoring tools such as GPS and VHT [96,97]. In South Africa, behavioral data from tracking systems, such as animals’ daily movements, behavior, and diet, are collected from collars fitted on animals [98,99]. Classification of some animal species behavior types, such as walking, eating, foraging, lying, standing, among others, are observed using GPS tracking and has scored an accuracy of over 80% [100]. The latter research shows that accelerometer data plus GPS tags contribute to a promising result in distinguishing more behaviors than could be classified with GPS alone. Adopting machine learning algorithms and integrating it into tracking systems to classify animal species behavior types while understanding brain–behavior relationships achieves acceptable accuracy. Their implementations using CNNs seek to understand five different behavior tasks in mice and humans through models that generalized to different behavior tests [101]. We note that both supervised and unsupervised machine learning methods help understand or monitor animal behavior. Some examples of this application are the prediction of animal behaviors from accelerometry data in pigs [102] and the classification regression decision trees used to track cows in open and habitat fields using differences in movement metrics between behavior types from GPS data [103]. In the latter, the GPS data with one-minute time step interval from the open field scored an accuracy of 70%, while data with 12 and 2 min time step interval failed to classify. These results show that the time step interval should be short enough for the behavior to be defined by its characteristic movement metrics. Moreover, data obtained from forested areas scored higher accuracy compared to an open field. Unsupervised algorithms are used extensively in identifying and modeling biologging and telemetry data such as hidden Markov models or Gaussian mixture models [104].

In summary, we have discussed the prevailing conditions that contribute to WVCs. In Section 3.1, we understand the contributions of roads and road features in reducing roadkill. Section 3.2 discusses how alterations in climate change and season boost animal mortality rates due to WVCs. Section 3.3 and Section 3.4 discuss the behaviors of humans and animals, respectively. To minimize these WVCs, we introduce Section 3 and discuss existing ADSs that prevent WVCs.

## 4. RQN2: What ADSs Are Deployed to Mitigate WVCs in the Primary Studies?

The existing intelligent technological systems used today to prevent WVCs are area-cover, break-the-beam, mobile mapping, buried cable, driver assistant, and autonomous vehicles ADSs. These operate independently, and some use machine learning algorithms to mitigate WVCs. Area coverage sensors and break-the-beam sensors are the two frequently used technologies to detect large animals within the range of the sensor either at a specific angle or in a linear shape as animals approach the road. These two systems are either active or passive [16]. Mobile mapping systems and roadkill detection systems detect small animals on the road. Buried cable systems can detect both the crossing of large- and medium-sized animals while providing data on their location along the length of the cable. Driver assistants and autonomous vehicles are the latest technologies deployed with machine learning models to help detect small, medium, and large animals. Even though these two seem promising in the field, the implementations are not exhaustive in developed economies, with less to no attempts made in developing and under-developed economies.

### 4.1. Area Cover Systems

For animal detection on the road, an area-cover system uses passive video sensors, passive infrared sensors, and active microwave radio sensors [105]. Table 4 identifies and describes each sensor type used by the area cover systems in this study.

An area cover system detects large animals in a distance of up to 50 meters within a $60^{\circ }$ horizontal angle [108]. The system operates by reacting to changing physical conditions that interfere with its electrical properties. They capture stimuli such as heat, sound, and movements that animals induce. These stimuli convert to digital signals and are then sent to a computer for processing and analysis. Area cover systems are passive or active. Passive area cover systems are composed of passive infrared and video detection, and active area cover systems are composed of microwave radar. These systems have successfully provided animals the liberty to move compared to the traditional fencing systems. However, these systems still face excessive false positives under vegetation cover [109]. In 2009, researchers developed and evaluated a test-bed composed of five cone-shaped area cover systems. These systems detect animals based on motion and body heat in an area and range from the sensor. Their records show a decrease in false positives and an increase in false negatives in passive area-cover systems due to high winds [18].

In 2003, the development and deployment of the thermal animal detection system (TADS) was successful. The TADS solved the intense collisions between birds and wind turbines in Denmark. To quickly achieve a reliable system for the TADS, thermal cameras are associated with hardware and software. These system recordings show birds migrating and nearing the wind turbine blades of the offshore wind farms. The system is sometimes effective under harsh conditions with poor visibility (fog, rain, or snow). A threshold temperature level is defined to ensure only sequences of birds observed and captured passing through the area cover system can eliminate false detections. The quality of the captured images are equally good in both compressed and non-compressed regimes. However, this work shows that collisions of turbines with birds are possible and highest at night when the visibility of the thermal cameras is poor [24].

The design and implementation of the flashing light animal sensing host (FLASH) in the U.S. helps detect deer on the U.S. Highway 30 pathway [25]. This system consisted of three data collection systems installed on this highway. These systems are the FLASH, warn, and motorist-speed systems. The FLASH system is composed of infrared sensors. These sensors trigger each time a deer moves through the detection area by emitting a signal to the receiving unit to activate flashing lights which are usually above permanently visible warning signs. The lights alert the drivers who are distant about the existence of a deer on the road. The intention is to prompt drivers to slow down the car’s speed determined by the motorist-speed system embedded with sensors. Next, sensors gathered data on vehicles on the highway before encountering the warning signs on either the right or the left sides of the road. Whenever the vehicles approach the detection area, speed sensors gather speed data on vehicles after viewing the sign and are already crossing. Moreover, the sensors register the number of axles of individual vehicles that pass and distinguishes them between automobiles and semi tractor-trailers. Further, the experts capture the time and date when each vehicle crosses the sensor. On manipulations of speed-sensor data and the FLASH data, the researchers are able to determine vehicle speed, vehicle type, date, time, and the status of the FLASH system (activated or not) for each vehicle. The data from the FLASH system is stored and downloads enabled remotely via modem. Results show that each factor significantly affects speed reduction for semi tractor-trailers under normal system operations. Factors, e.g., date, had a probability value equal to 0.003. For the time of day, the probability value is less than 0.001, and treatment had a probability value of less than 0.001. Automobiles also had a significant speed reduction for each factor, e.g., time of day had a probability value less than 0.001 and treatment had a less than 0.001 probability value. The date had no significant impact on the automobile’s speed because the date had a high probability value of 0.101. Some months influenced vehicle speed more than others. During December, semi tractor-trailers reduce their speed to 1.1 km per hour more than during January, February, and March. The vehicles reduce their speed to a minimum in the daytime and relatively high at night each day. This happens when the lights are either activated or deactivated. For experimental treatments, results show that all factors are significant with automobiles having a probability value of less than 0.001, and semi tractor-trailer records treatment and time of day as significant. Results regard date as not significant since it had a high probability value of 0.247. However, even when the system results are favorable, the system is unreliable for the U.S. Highway 30 pathway. This system can still operate well on roads with a huge percentage of local traffic than the U.S. Highway 30 pathway. In improving the effectiveness of the U.S. Highway 30 pathway at reducing deer–vehicle collisions, a few recommendations are suggested, such as the installation of a deer-proof fence as well as implementing an education program for the local motorists explaining how the system operates.

In 2006, the Oh DEER, Inc. (Wayland, MA, USA) company developed an area-cover system which detects white-tailed deer (large animal) within a certain range of a sensor [17]. These sensors transmit and receive microwave radio signals to detect large animal movements. The system stores detection data such as identification number, type of sensor, detection value, whether the flashing lights are turned on or not, battery voltage, solar panel voltage, and temperature in the box with equipment on a flashcard. The last three parameters are allowed for identifying potential problems. The data are downloadable on-site or remotely through a modem and land-based phone line. The remote location system configurations can enable time delay for a signal turn-off. In 2013, Mukherjee et al. [27] developed an area cover system by integrating both passive infrared and short-range. and installed the system comfortably in terrain landscapes. Their system monitors animals within the virtual trap wire. Further, their system experiences high false alarms during the day, false detection, detection delay, and difficulty in detection during bad weather.

In 2017, a sensor composed of Doppler radar technology is used in detecting large animals in a detection area and alerting drivers through activated warning signs [23]. The focus is to achieve the reliability and effectiveness of the ADS. In evaluating how reliable radars are in detecting large animals, they adopted thermal cameras in monitoring animals at the area coverage. Each time doppler radar detected an image of a large animal, the thermal camera captures images every three seconds and saves them temporarily. To measure the effectiveness of the systems in reducing vehicle speed, they install speed radars to record the speed of individual vehicles. The radar records speed between the northbound and southbound lanes as vehicles approach the detection area. Evaluation of the reliability and effectiveness of the system is through conducting tests such as walk-through tests and seasonal reliability tests. Even though the researchers consider this system reliable and effective, it still registers false positives and negatives. In the same year, Vikhram et al. [26] developed an ADS in the farm areas to prevent human–wildlife conflicts. They used a passive infrared sensor to detect animals’ movement by sending a signal off the controller. In addition, they also use ultrasonic sensors to generate a sound sent by a global system for mobile communications to alert the farmer and department through messages about the presence of an animal. Conducting system testing under bad weather was successful. Doppler radar is used again in the detection of animals and humans under vegetation cover in 2018 [22]. The Doppler radar has an HB100 sensor used in transmitting and receiving microwave radiation. If the strength of the reflected signal is weaker than the one initially sent, then the radar will invoke a detection. The Doppler radar is placed and moved in front of the different crops. In addition, a dog or person is placed behind the crops. Under this situation, the radar is able to detect these animals. Detection of humans is in five out of seven cases and a dog in two out of seven cases. Although doppler microwave radar detects animals, we need more research to minimize the disturbing factors on the measurements.

We summarize area cover systems in Table 5 while showing the different sensor types, performances, strengths, and weaknesses.

### 4.2. Break-the-Beam Systems

Break-the-beam systems use transmitters and receivers with infrared, laser, or microwave radio signals. When an animal is present within the system’s beam, a warning sign is activated. We describe break-the-beam ADSs currently deployed around the globe showing their strengths and weakness.

In the last decades, the number of WVCs in North America was alarming. These came with negative effects, prompting researchers to develop a break-the-beam system to mitigate roadkill [17]. The system detects large animals as they approach the roads by activating warning signs to alert drivers about the existence of animals on the road or in a near distance. The sensor technologies and systems experts—a vendor company—conducts field tests. The designing of the break-the-beam system is by the vendor company. The system consists of a transmitter that sends modulated signals to the receiver. Suppose an animal breaks the paired sensor beam. In that case, activated warning signs trigger an alert to the driver about the existence of an animal on the highway which prompts the driver to reduce the speed. Increase in alerts leads to fewer or less severe carcasses with animals. They save the recordings of all detection at a master station. Detection events are broadcast in real-time using the ultra-high frequency radio system. The detections enabled on-site monitoring of the ADS operation using a portable data radio connected to a laptop computer. The study saves every change in beam status at every date and time. However, this system has drawbacks such as (1) covering longer distances, which requires many sensors, hence making the system expensive, and (2) the system requires more hardware resources on curves, slopes, and vegetation landscapes.

In 2009, an installation of a test-bed of four break-the-beam systems are successful in detecting large animals [18]. These systems consisted of infrared, laser, or microwave radio signals. Most of these systems had two sensors on a pole in the middle of the 91 m distance, with the sensors facing opposite directions. On evaluating the systems, the researchers achieve excellent visibility associated with fewer false positives. However, these systems also experienced challenges such as (1) high error rates due to high temperatures; this is due to the failure of infrared sensors failing to differentiate between hot air and body heat of animals, (2) terrains are not suitable for the installation of break-the-beam, and (3) high false detections due to thick vegetation cover. In California, along Highway 3, engineers installation of a microwave break-the-beam ADS was successful the next year. The system site is used as a test-bed to determine factors to consider when selecting an ADS, and it also shows the reliability tests of the system [20]. The test-bed is composed of an animal enclosure, space for many ADSs, and six infrared cameras with recording capabilities. The animal enclosure included shelter, water, area for feeding. This system detects animals, records the date and time of each detection, and further records animal movement within the enclosure. This work investigates the reliability of the system. The reliability test results show that the percentage of false positives is 0.007% (1 false positive over 140 valid detections), false negatives is 0.03% (4 false negatives over 140 valid detections), and 148 intrusions in the detection area, of which the study shows 144 detections, which led to 97% detections of intrusions in the detection area. Although these system results show high detection rates, it still experiences false negatives related to the shortest animal, “sheep”, in the enclosure. As a technique of eliminating the false negatives, we proposed cutting the grass low in the enclosure.

In 2016, installations of a roadside animal detection system (RADS) in Florida [21] was successful in helping to reduce WVCs that are rampant. The system uses an infrared beam to initiate communication between transmitters and receivers. A paired combination of these transmitters and receivers communicate through sending and receiving signals. When an animal crosses between the sensors, the light beam sends back a weaker signal. Each time a sensor receives a weak signal, a flashing light turns on, warning the drivers that an animal is on the highway. The system performance result is average in detecting target species. The average success rates were between 10.7 and 66%, and average failure rates were between 34 and 89.3% of target animals not detected, resulting in false negatives. Generally, 90% classifications of RADS activations are false positives (lights flashing and animals not present). These activations cause a significant reduction in vehicle speed. The effectiveness of RADS is also tested in a controlled setting using a driving simulator. Driver simulation produced positive results such as (1) quick reductions in speed when exposed to picture-based RADS signs than word-based RADS signs, (2) early braking in response to observations of an animal in the road, (3) relatively low WVC cases, and (4) picture-based RADS signs create alertness of drivers more than word-based RADS signs. These tests show that the system is reliable and effective in detecting large animals and improving motorcyclists’ road safety. However, the system still experiences downfalls such as false detections, malfunction, and drivers’ ignorance of the usage.

In 2019, Antonio et al. [19] simulated an ADS that detects animals on the road and alerts drivers. The system is composed of camera modules with microwave sensors and computing units. The type of sensors used in their system are the break-the-beam sensors. The computing unit is composed of a low-cost server, a management system and alert processing, a warning signal on the road, and network equipment (a switch) that makes the interconnection between all of these devices. Further, this architecture contains two cameras, together with two sensors, which capture an animal’s image and send it to the management system. This system initiates the machine learning algorithms to detect and classify these images of moving animals. If an animal faces danger due to cars, the particular animal is detected on the road by the management system, which alerts the warning sign on the road for the driver to be cautious. This proposed simulation is the first to integrate the concept of machine learning algorithms in detecting a specific or target species. Among all supervised algorithms used, KNN shows the best F-measure. However, this work also faces downfalls such as using synthetic images for training the model. The work also experiences less accuracy, with failure in detecting small animals. Moreover, the study is characterized by fewer data with no road tests performed during the testing phase.

It is noticeable that break-the-beam systems effectively detect large animals, which then reduces the collision of large animals and vehicles. The most recent break-the-beam systems are compared and summarized in Table 6. The systems have also registered false positives due to sensors’ movement and low visibility. Due to high rates of false positives, drivers attempt to ignore the road’s warning signs, mistaking the system to be facing a malfunction. Integration of advanced machine learning algorithms can help make the system intelligent and eliminate false positives, achieving higher accuracy. Equally, adopting the use of state-of-the-art machine learning approaches in RQN4 and integrating them with sensor technology can minimize the false detection and increase the accuracy of the system.

### 4.3. Buried Cable, Roadkill Detection, Driver Assistance, Autonomous Vehicles, and Mobile Mapping ADSs

Installations of buried cable animal detection systems (BCADS) for wildlife–vehicle detection were in the fall of 2008 along U.S. Highway 160 between Durango and Bayfield [28]. These systems can generate an invisible electromagnetic field that faces disturbance each time a large animal crosses the cable. The disturbance has an adjustable threshold above which it triggers an alarm and activates warning signs, cautioning drivers to be more alert and reduce vehicle speed to help mitigate collisions of cars and ungulates. In 2009, installations of cameras to capture the image of animals crossing the road during both the day and night were successful. The records of images are associated with references such as the date and time stamp. The results of the reliability investigation of the system show ∼0.46% of false negatives with no false positives. The percentage of detections of all intrusions in the electromagnetic field (detection area) is relatively high, i.e., ∼98.12%. When tested under different environmental conditions, the system generates no false positives and approximately 0.46% false negatives. In 2015, Christian et al. [29] installed a BCADS. The system detects large- and medium-sized animals crossing the road and provides data on their location along the length of the cable. The system generates an invisible electromagnetic detection field around the buried cables. The components of the system are a 300 m long-buried dual-cable sensor and a central processor unit for control and communication which are similar to works by Huijser et al. [28]. In their study, each time the area where detections happen are perturbed, an alarm is declared, and they determine the location of the intrusion. Their work has a surveillance video camera which monitors animal movement and gauges system detections during nighttime and under all-weather conditions. Results show that if properly installed and calibrated, BCADS can detect large and possibly smaller animals with over 95% reliability. However, this system still registers false detections.

In 2016, mortality rates of amphibians on Portuguese roads had been rampant [35]. Installation of a roadkill-intelligent system successfully scans for the dead amphibians, hence capturing roadkill records. This system scans the road using a trichromatic line scan camera with light-emitting diode lighting. The two-axle trailer is attached to another vehicle such as a lorry jeep, among others, and pulled. The architecture of the system is composed of an imaging system used for acquiring road surface images: a computer used to control the device, storage, and processing unit that can even operate offline, a GPS for extracting the coordinates for all acquired read frames, a line scan camera and light-emitting diodes used for capturing images, and an encoder used to synchronize all the device. Some of the criteria to follow on implementing such a system are (1) mobility—easy and free system movement, (2) versatility—that is, the flexibility to accommodate all vehicles, (3) motion stability needed for the image capturing system and thus the dual axle system, (4) standalone powering over several hours of utilization, (5) accommodation of all the equipment with protection to rain and dust, and (6) capability to scan the road surface with high resolution of a complete track (∼25 km) at an acceptable speed. However, even though such excellent criteria is followed, this system still experiences downfalls such as the lack of machine learning algorithms to automatically detect roadkill of amphibians with high accuracy.

In 2017, Sharma and Shah [31] developed driver assistance to detect animals on highways. Their system uses the histogram of oriented gradients descriptor and cascade classifier to detect videos and images with less computing space. As such, the system achieves a high accuracy due to the ability of cascade classifiers to remove false negatives quickly from the data samples. The algorithm achieves an 80% accuracy when using a small sample of dataset available. Their system does not capture and test the images during the night. Even with these weaknesses, the algorithm accurately detects video and images on the road and alerts the driver whenever the algorithm detects a cow. In the same year, Rosenband [32] discussed the implementation of a self-driving car that requires no human. The car uses software and hardware components to automate navigation. The software helps to simulate and analyze vast amounts of datasets to make self-driving cars a reality. Besides, the hardware contributes to automation through sensing, computing, and embedded control with the help of the software. The sensors collect information such as vehicle surroundings, position, and environment. Later, the sensors send the collected information to a computing unit for processing. The computing unit fuses, processes, and interprets the sensor data. The output of the computing unit is the trajectories. Next, these trajectories pass to embedded control systems, which in turn communicate with the vehicle actuators to manipulate steering, braking, and throttle. The dataset used for detection is images and videos. However, this study is limited with animal datasets, limiting the performance of autonomous vehicles in detecting WVCs. It is worthy to note that less research is conducted on mitigating WVCs using autonomous vehicles.

In 2018, Sillero et al. [33] developed a prototype of a mobile mapping system for classifying amphibians on the road. This system is composed of a scanning system attached to a vehicle. The scanning gadget can record amphibian images on road surfaces up to a speed of 30 km per hour. On evaluating the system, they conduct three tests which show promising results. The first test controls are accompanied by plastic specimens; the second control tests are composed of dead specimens. In addition, the third test is composed of real tests on Mitra campus roads. The data collection process performs analysis and testing on amphibian datasets, achieving acceptable results. The first test correctly classified 94.1%, and failed classification rate is at 5.88%. The second test correctly classified 92.3% dead specimens and failed to classify 7.7%. Finally, with the third test, 83.9% real specimens are classified correctly, and 16.1% failed to be classified. In all cases, the human classification of images achieves 100% correctness. This study is the first to use machine learning algorithms and conduct road tests to detect live and dead amphibians. This work can adapt to any animal group. Unfortunately, the algorithm presented a high rate of false-positive detections.

In 2019, development of a smaller and improved version of the mobile mapping system is successfully implemented in Portugal [34]. It detects dead amphibians and small birds on the road. The new system components include a ZED stereo camera (stereo multi-spectral and high definition camera), a GPS receptor, a high-power processing computer (GL553VD ASUS laptop), an attachment device, and a lighter charger for vehicles. The manufacturer of the camera component Stereolabs Inc is located in San Francisco, CA, USA. Whereas that of the GL553VD ASUS laptop is ASUS company whose headquarters is at Taipei City, Taiwan. The system runs continuously as long as the vehicle is working. This novel solution is advantageous because of the reduction in the system size, hence being light weight, making it portable and easily attached to the back of any vehicle. Other advantages of this system are more straightforward workflow, unlimited energetic consumption, and sampling width which effectively covers a one-way national road, and the system is considerably cheaper. In measuring the effectiveness of the system, the system detects approximately 78% of the amphibians and birds present on surveyed roads, failing to detect 22% and generating approximately 17% of false positives. This system is applicable and feasible and can improve the implementation of conservation measures. The system saves time for researchers and transportation planning professionals. In the same year, Goswami et al. [110] started developing deep learning algorithms for driver assistance to detect animals and further proposed a dashboard framework to deploy algorithms. The dashboard systems are usually portable and efficiently assist the driver by generating a message signal whenever they detect an animal in the camera frame. Even though this study had a limited dataset, results show that MobileNet-SSD outperforms Mask R-CNN and YOLOv3 in detecting objects.

The Virginia Department of Transportation, in collaboration with the Virginia Tech Transportation Institute, participated in installing BCADS. The system acts as a measure to mitigate the WVCs [30]. The systems are powered by a solar photovoltaic system and a cellular modem provided for remote system monitoring and data collection, installation of a flashing light—“deer crossing”—warning sign at the site, and wirelessly linked to BCADS to alert approaching drivers whenever a detection of an animal is observed. These systems provide roadside or in-vehicle warnings if an animal is active on or near the road. The system’s performance is monitored on a public road to assess real-world system capabilities and operational issues. On evaluation under controlled and secure conditions, there was successful reliability and effectiveness of BCADS. These systems identify crossings of large- and medium-sized animals with approximately 99% reliability and provide data on their exact location along the length of the sensing cable. Under all-weather conditions, BCADS collected data to monitor animal movement, vehicle traffic, and system performance. The authors collect data on driver response; warning signs are activated during the dawn and dusk hours, and the data shows that approximately 80% of drivers either braked or slowed in response, indicating that the signs are effective. However, we also see that 20% of the drivers are not complying with the warning signs.

In summary, although existing intelligent ADSs detect large animals, these systems still register a lot of false detections. Generally, the systems fail to distinguish between the movements of large animals and other objects. Besides, they fail to restrict detections to only wildlife movement. With this, these systems require algorithms that will help them distinguish between different objects or help recognize target objects, hence reducing the errors that lead to false positives and false negatives. Moreover, todate, there is no work that uses geophones, UAVs, GPS, and VHT tags to detect animals with a motive of mitigating WVCs. Table 7 summarizes the buried cable, roadkill detection, driver assistance, autonomous vehicles, and mobile mapping ADS. In Section 5, we discuss the different datasets used to prevent WVCs.

## 5. RQN3: What Types of Datasets Are Currently Used to Mitigate WVC?

In this section, we provide a survey of the available datasets used in the primary literature to mitigate WVCs. Moreover, the section suggests datasets to use in mitigating WVCs. For each of the datasets, we identify different tasks implemented in the primary studies to mitigate WVCs, and we propose additional tasks to apply to the dataset to further minimize WVCs. Data collectors such as citizen scientists, experts, and Kaggle collect the identified datasets. Table 8 shows parameters captured and these are name of dataset, type of data, task executed or proposed, collectors, data collection period, the number of instances, country, and the reference works. The datasets in Table 8 are presented in alphabetical order and are composed of time series, image, and sound datasets. The label 🗸 indicates that the work at the “Ref works” column has implemented the specified task. The symbol × means a specific task is proposed to be implemented with the dataset, but we did not find any work that implements the task in the literature. The label − indicates that the authors are not explicit about a specified parameter value in the column. We note that we did not find reference works for rows labeled as “None”.

The time series datasets are major records of WVC instances. For time series, the collectors capture the name and the number of species killed in a specific time frame. Datasets such as WVC hotspots [112] and snake roadkill [39] predict WVCs. Possibilities of other machine learning tasks that may use these two datasets are classification and forecasting. The WVC dataset [112] contains a count of 529 species killed in Italy. The data are collected from 2014 to 2016 by citizen scientists. Predictions of WVCs are observed by Valerio et al. [112] while using this dataset. In Taiwan, citizen scientists collect and record ∼40,000 instances of snake roadkill [39]. Later, the dataset helped Yue et al. [39] to predict the death of snakes on the road. The collection period is from 2006 to 2017. It is worthy to note that time series datasets such as animal-related crash data [113] and roadkill datasets [114,115,116] all focus only on collection but never perform any machine learning techniques on the data. Animal-related crash data [113] are collected by experts from 2001 to 2007. Every year, 0 to 4285 instances are collected in Australia. From 2007 to 2008 in Kenya, data on roadkill [114] are collected. The collectors record 1436 instances for 164 days over an 11 year period. We find no experiments performed on this dataset. Therefore, implementation of machine learning models is vital to perform experiments. Besides, in Tanzania, roadkill data [115] collections happened between 1990 and 1991. The experts collected 183 instances. In South Africa, the roadkill dataset collected from 2011 to 2014 helps mitigate WVCs [116].

Image datasets such as Pothole dataset [117], Stanford cars dataset [118], Oregon wildlife dataset [119], and Kangaroo dataset (https://www.kaggle.com/hugozanini1/kangaroodataset, accessed on 14 August 2021) are vital in mitigating WVCs. These majorly capture examples of the different animal images used in a machine learning model or framework. They perform object detection tasks with other tasks such as classification, segmentation, and localization not exhaustively applied. Fan et al. [120] use the Pothole datasets [117], Oregon wildlife dataset [119], and Stanford cars dataset [118] in their study to mitigate roadkill. The latter dataset detects and distinguishes vehicles from other objects. These three datasets are collected on different occasions and in different locations for the use of object detection. In 2018, in the USA, a dataset composed of a collection of cars was collected by Kaggle and referred to as Stanford cars dataset [118]. It is a large dataset with approximately 16,185 records of car images. The Oregon wildlife dataset [119] is collected from Oregon in 2019. The dataset is open source and published on Kaggle for easy access. It contains 2029 image instances. In the same year, 2019, Pothole image dataset [117] is collected by scraping online images.

Additionally, object detection is further being applied to Kangaroo dataset (https://www.kaggle.com/hugozanini1/kangaroodataset, accessed on 14 August 2021) and Serengeti dataset (https://lila.science/datasets/snapshot-serengeti, accessed on 20 September 2021). In 2020, 313 images of Kangaroo are scraped online to collect a Kangaroo dataset (https://www.kaggle.com/hugozanini1/kangaroodataset, accessed on 14 August 2021). Later, the images are annotated to build machine learning pipelines for object detection. Object detection achieves tremendous results on this dataset when applied by Yi [41]. In addition, other machine learning tasks such as classification, segmentation, and localization may apply to this dataset. Another fundamental dataset is the Serengeti dataset (https://lila.science/datasets/snapshot-serengeti, accessed on 20 September 2021) which contains 7.1 million images collected by conservationists in 2010 in Tanzania. It contains a variety of animal species used to mitigate WVCs when modeled with machine learning. Some works by Marco et al. [121] and Sadegh et al. [122] design object detection algorithms that detect and count animals in wild reserves. The image dataset YOLOv3 achieves high accuracy on an image localization task by Redmon et al. [123]. Moreover, the dataset contains images of animals used to mitigate WVCs. In 2020, 1000 instances of Cheetah, Hyena, Jaguar and Tiger dataset (https://www.kaggle.com/c/swdl2020/data, accessed on 1 September 2021) are collected by experts. This dataset has a good sample size, prompting us to recommend tasks such as object detection, localization, classification, and segmentation.

Other image datasets such as YOLOv3 (https://pjreddie.com/darknet/yolo/, accessed on 9 December 2021) and Waymo open dataset (http://www.waymo.com/open, accessed on 11 December 2021) have performed with acceptable results in localization, and detection and tracking tasks, respectively. YOLOv3 (https://pjreddie.com/darknet/yolo/, accessed on 9 December 2021) dataset used localization techniques [123] to mitigate WVCs. Waymo open dataset [124] (http://www.waymo.com/open, accessed on 11 December 2021) is a state-of-the-art image latest image dataset for autonomous driving. The dataset contains 1150 scenes, each of which spans 20 s. It is composed of images of calibrated high-quality LiDAR and camera data. The tasks that use this dataset are detection and tracking.

Sound dataset such as wildlife–train collisions data [125] are vital in mitigating WVCs. This particular dataset is collected from different sensors whenever a train approaches a hotspot area. The train uses an approach detector which uses vibrations in the rail to detect trains and creates a warning system. When the warning activates, animals leave the railway, hence mitigating roadkill cases.

We find that some state-of-the-art datasets, such as those used in autonomous cars, do not capture animals as a sample in the training examples, e.g., Kitti dataset [126]. Because of this, there is a need for a balanced, fair dataset for detecting animals. This balanced, unbiased dataset increases the accuracy of detecting animals in autonomous vehicles’ ADSs. Other systems such as break-the-beam, area-cover, and driver assistance can embrace such image datasets highlighted in Table 8.

## 6. RQN4: What Types of Machine Learning Algorithms Are Used to Mitigate WVCs?

In 2013, Parikh et al. [127] used MATLAB to implement template matching and motion detection using the frame difference method to detect birds on the road. While detecting the motion of objects in the video, denoising for the frame and several frames is initiated on a two-frame difference. Additionally, researchers perform background subtraction to obtain a binary image [128] and subtract two frames to attain differencing. These subtractions are mostly performed pixel wise. This system operates by using stored video and background subtraction methods. Features extraction is from the stored video and database. Later, the features generate target and template images which are fed into a template matching algorithm for detection. Template matching is a technique in digital image processing for finding small parts of an image that match a template image. While implementing template matching, authors adopt the concept of normalized cross-correlation. On measuring the similarity of the images, the results varied from −1 to 1 with 1 meaning that the image is identical, −1 that the image is the negative of the other one, and 0 that the two immediate images do not correlate. When the bird is detected, a generated alarm triggers as feedback. On testing, the background subtraction methods perform well in detecting birds. The false-positive and false-negative for bird detection are 5.94 and 5.23%, respectively. This technique had 94% efficiency in detecting birds.

In 2017, Tibor et al. [38] created an animal dataset that consists of 500 different subjects (5 classes with 100 images for each class). The five classes are bear, hog, deer, fox, and wolf. Recognition and classification of the different animal species using CNN are successful. Other algorithms compared with CNN in recognition of animals are those such as: (1) principal component analysis (PCA), in which the PCA reduces the dimensionality of large datasets by transforming a big set of input variables into a smaller one with almost all the information obtained in the big set present [129]; (2) linear discriminant analysis (LDA), which reduces the dimensionality of the data by maximizing the separability among known patterns [130]; (3) local binary patterns histograms (LBPH), an image recognition algorithm that uses pixel’s 3×3 neighborhood to extract the LBP code and in which the process involves thresholding the 3×3 neighborhood of each pixel with the center value using mean, median, and center pixel as thresholds [131]; and (4) SVM which classifies the different species. Results show that the CNN model achieves the best accuracy of 97% on a bear, 95% on a wolf, 95% on a fox, 93% on a deer, and 91% on a hog. The highest accuracy scored by other models is 82% on bear and hog by PCA, 83% on a hog by LDA, 87% on wolf by LBPH, and 87% on a bear by SVM as summarized in Table 9. Even though this study did not test this application on the roads, we highlight that the work is vital in recognition and could classify animals on roads when integrated with an alert system to minimize WVC.

Antonio et al. [19] developed a methodology that detects animals using animal images captured by roadside installed cameras. This methodology allows features extraction of regions of the image and further uses machine learning techniques to classify areas into two classes: animal and non-animal. In this work, the KNN and random forest (RF) algorithms achieve acceptable generalization. KNN ranks a test sample according to the class of its *K* nearest training samples and also defined according to a distance metric. RF generates randomness for a model when the trees grow. Instead of finding the best feature of splitting a node, it looks best among a random subset of resources. Results show that KNN had a slightly higher accuracy of 0.6243 when compared to RF with an accuracy of 0.6219. However, adoption of more robust machine learning models is vital to help achieve accuracy greater than 0.6243.

In 2019, mobile mapping system 1 was upgraded to mobile mapping system 2 in Portugal [34]. Mobile mapping system 2 includes state-of-the-art deep learning image classifiers such as (1) VGG16, a CNN model with a 16 weight deep layers network of a stack of convolutional layers, fully connected layers, softmax layers, and rectified linear activation function [132], (2) VGG19, which contains 19 weight network layers of 16 convolutions and 3 fully connected layers, in which an image passes a stack of convolutional layers, max-pooling layers, fully connected layers, softmax layers, and rectified linear activation function to be recognized [132], (3) ResNet50, a 50 layer residual network stacked and trained for a particular task, it learns many features at the end of its layers using skip connections, and the residual is a subtraction of a feature learned from an input of a layer [133], (4) Inception-V3 which modifies the previous inception models by using less computational power [134], and (5) Xception, a deep CNN architecture that depends on depthwise separable convolutions layers. Depthwise separable convolutions are composed of depthwise and pointwise convolution [135]. These image classifiers tested on dead amphibians and birds have achieved tremendous results. Real and controlled surveys help to evaluate the system’s performance. Controlled surveys use a dataset of the dead specimen from the University of Evora, and researchers conduct real surveys on the national roads of Evora in real-time. In real surveys, detecting dead species is approximately 78% of the amphibians and birds on Evora roads, with a false negative of 22% and roughly 17% of false positives. Controlled surveys detections are between 63.3 and 80% of animals. The false positives are between 17.4 and 24% and false negatives between 20 and 36.7%. In East Asia, mortality rates of different species of snakes dataset are citizen-collected from Taiwan [39] as shown in Table 8. The maximum entropy environmental niche modeling helps in predicting the snake roadkill. This method finds the probability distribution that best fits data [136]. Results show that patterns across snake species differed in habitat use, foraging behavior, and taxonomic group. Roadkill sightings are highest in forests and strips of farmland or shrubland that cut through forests; these areas likely support high snake abundances or dispersal activity. Sightings are lowest in urban areas, likely because of unfavorable habitat conditions. Road density has little influence on roadkill sightings. Dense roads may be associated with lower habitat quality, hence containing fewer snakes. To achieve high accuracy in mitigating snake roadkill efficiently, considerations of natural history and landscape factors are vital.

In 2020, a dataset composed of various image samples is split into 75% train and 25% test and trained on a CNN vision algorithm [40]. The vision algorithm detects and classifies a cheetah at 79% accuracy and an elephant at 86% accuracy. It uses a four-layer approach to train the input images, e.g., convolutional layer that extracts features from an input image, rectified activation function, pooling layer, and fully connected layer. In the same year, Yi et al. [41] innovated and developed a roadkill alert system called ROOD. This system alerts drivers of high-risk areas before they encounter animals. It does this by detecting dead animals on the road and sending signals to drivers by using LED lights and vibration to detect the roadkill. If the driver detects the roadkill as a true positive, the driver sends the GPS location and photos back to the database. The reported GPS location sets up an instant alert to the nearby drivers, and the location is marked a high-risk area based on the collision rate. The dataset used in this study is YOLOv3 (https://pjreddie.com/darknet/yolo/, accessed on 9 December 2021) and Kangaroo dataset (https://www.kaggle.com/hugozanini1/kangaroodataset, accessed on 14 August 2021) for training the YOLOv3 algorithm [123]. The algorithm uses residual block, bounding box regression, and intersection over union techniques. The image is divided into many grids of equal dimensions for the residual blocks. Every grid cell will detect objects that appear within them. For example, if an object center appears within a specific grid cell, this cell will be responsible for detecting it. The bounding box outlines and highlights an object in an image. The bounding box attributes are width, height, class (for example, lion, elephant, etc.), and bounding box center. YOLOv3 uses a single bounding box regression to predict objects’ height, width, center, and class. Later, the algorithm represents the probability of an object appearing in the bounding box. Intersection over union is a metric in localization that describes how boxes overlap. Each grid cell is responsible for predicting the bounding boxes and their confidence scores. High performance is when the intersection over union is equal or near to 1, and poor performance is when the intersection over union is equal to 0 or closer to 0. The study allows road authorities and biologists to respond to the collisions immediately and assists in planning road infrastructure improvements. This system was not installed on real cars so that each vehicle turns into a peri-urban citizen. Adequately, this research suggests three potential directions for mitigating roadkill: changing animals’ behavior, improving road infrastructures, and changing driver behavior.

**Figure 6:**
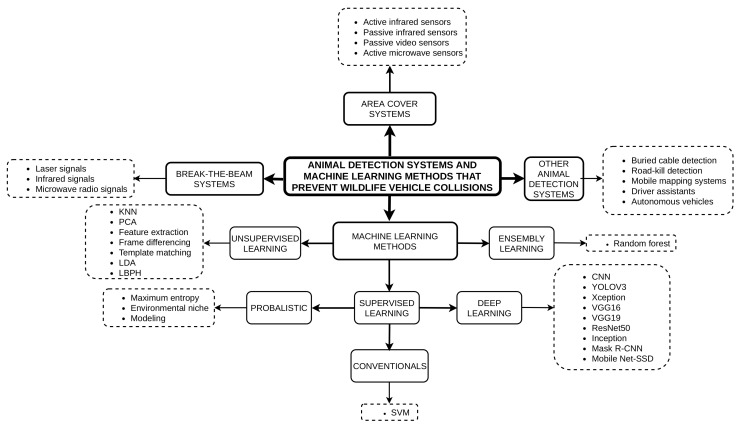
Summary of existing intelligent systems and/or machine learning methods that prevent WVCs.

In summary, this section discusses works that use machine learning algorithms to mitigate WVCs as shown in Table 10. Figure 6 captures the intelligent systems and the different tools and devices integrated into these intelligent systems to mitigate WVCs. We also note in Figure 6 the various types of machine learning algorithms currently implemented to detect animals and to mitigate WVCs. Moreover, we find that most machine learning algorithms used to detect animals in wildlife do not mitigate WVCs but monitor and count species. Besides, there is non-exhaustive research on mitigating WVCs using reinforcement learning agents. As well, no identified research is exhaustive on the integration of machine learning with ADSs such as area cover and BCADS. In Section 7, we discuss issues arising from the review and suggestions on filling in the gap.

## 7. RQN5: What Are the Limitations of the Reviewed Studies in Mitigating WVCs? What Are the Proposed Solutions?

Many attempts highlighted in Section 4 and Section 6 minimize WVCs. To date, roadkill continues to be rampant due to prevailing conditions in Section 3 that contribute to the occurrence of WVCs. Moreover, implemented solutions discussed in Section 4 and Section 6 are not effective in reducing roadkill due to high rates of false detections. We highlight, in-depth, the issues and challenges arising from the review and the way forward as summarized in Table 11.

### 7.1. Current Issues and Challenges Arising from the Review

Although these systems record tremendous results in mitigating WVCs, they still experience enormous issues and challenges:

- Most current ADSs are not suitable for small- and medium-sized animals. We note that small animals are killed more by WVCs than big animals, which requires urgent attention in implementing tools that mitigate WVCs for all animal species. Most solutions concentrate on creating solutions that detect large animals, leaving the non-large animals vulnerable to roadkill.
- The machine learning algorithms do not understand each animal species and its behaviors but generalize species. Developing machine learning models that understand and detect animal behavior of each species is vital.
- There is high false detection due to bad weather conditions and thick vegetation cover, among other factors. The detections are either false positive detections (where the system reports a detection and there is no animal) or false negative detections (where the system did not report any detection yet there was a large animal present) or both.
- Some current systems fail to create an alarm to alert the driver whenever the system detects an animal. In addition, other systems experience delays in detection and sending alerts.
- The embedded solutions implemented so far are usually expensive. There is no low-cost hardware that mitigates WVCs.
- There is a gap in the investigations of fairness in Artificial Intelligence (AI) [137], without excluding AI for the wildlife conservation sector [138]. Auditing and explanation of machine learning algorithms is vital to ensure algorithms observe fairness in machine learning.
- ADSs do not exist in South Africa and Africa and mostly exist in the Northern Hemisphere.
- Limited datasets for training machine learning models to detect animals on the road.
- Mobile mapping systems need human intervention to detect dead amphibians and birds. Moreover, the system captures poor quality images during bad weather seasons.
- The ignorance of drivers and other road users on the usage of the existing ADSs greatly limits systems to achieve acceptable results during system testing.

### 7.2. Future Research Directions and Way Forward

**Figure 7:**
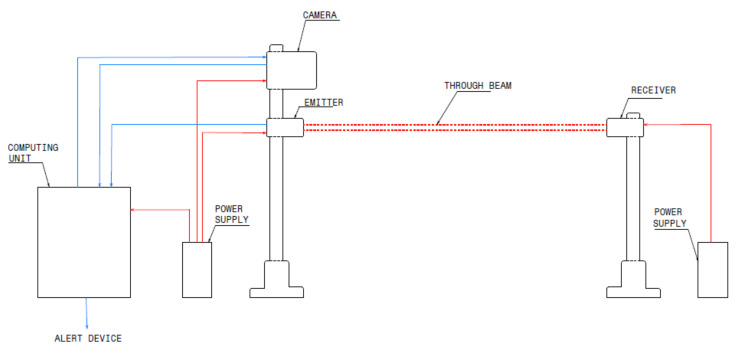
A proposed real-time roadside ADS with a computing unit that has machine learning models to detect animal species captured by the camera unit.

Our review being investigative and interpretive raises opportunities for future research directions. It does this by identifying challenges in Section 7.1 and then proposes future research directions. Implementing the proposed solutions as stated in Section 7.1 depends on the demands of new learning environments. In this section, we discuss the future research directions as listed below;

- We suggest the design and development of a matching algorithm(s). The algorithm(s) should match species to season(s) or month(s) of the year when vulnerable identified species make frequent crossing attempts toward the opposite side of the road. It is worthy to note that different animal species possess different behaviors during different seasons. Because of these fluctuations in animal behavior, each animal species will frequent the roads during different times and seasons. Animal movement dataset is vital when designing the matching algorithm. This is because the dataset records location coordinates, animal name, sensor type, and timestamp [139]. Further, drivers can be sent alerts each time the algorithm matches a specific species to season(s) or month(s). The notifications should tell the driver that there is a high probability that a specific species is on the road or near the road. The alerts can be sent on the mobile client or dashboard through GPS. Besides, these machine learning algorithms can use factors such as climate to alert the expected presence of animals toward roads during a specific period of the year. Proposal of using a movement dataset is possible because this kind of dataset, together with machine learning models such as hidden Markov models, has shown promising results in classifying animal behavior and estimating the location of species by GPS telemetry [140].
- We recommend upgrading or updating existing systems. The existing systems may be modified and integrated with cutting-edge improved vision algorithms to detect each animal species and classify it with classical animal monitoring system capabilities. Such a solution will increase the accuracy of the already implemented systems in preventing WVCs. Most of these current systems implemented are either break-the-beam, area cover, or buried cable ADSs using sensor technology. However, combining these sensor technologies with vision algorithms is vital and helps detect specific individual species, hence minimizing the false detections.
- We propose development of a real-time roadside ADS as shown in Figure 7 in Africa. This system should monitor and track animals on the road. It is composed of (1) a power supply, supplying the whole system with power. It supplies the computing unit, camera, and sensors, (2) a switch to connect all the hardware devices or system, and (3) photoelectric sensors having an emitter and receiver. The emitter sends a beam of light to the receiver. When an animal crosses the light beam, the receiver alerts the emitter about the presence of an animal. It also includes a (4) camera—when the emitter sensor is alerted of a presence of an animal on the road, it invokes the camera to take the picture. On taking the picture, the camera saves this information to the computing unit (5) raspberry Pi, which is configured together with machine learning algorithms to detect animals on the roads. The computing unit should be configured with protocol buffers or Python to enable system interconnection. As well as communication between the hardware and software and (6) warning signal device (LED-matrix display), the device is placed on the road for the drivers to read and be notified if an animal is crossing the road. When the picture of an animal is taken by the camera, the computing unit processes the images using machine learning algorithms. The LED-matrix display sends an alert on the highway if it detects the image. The system can further be implemented as a reinforcement learning agent to operate like the real-time roadside animal detection proposed system.
- We suggest developing other new, improved, and efficient solutions. The solutions should be different from the real-time roadside ADS proposed above; such solutions can be, e.g., (1) a system that extends to send an alert to police in case of a collision to clear up the road. The notification can be a text message or a mobile application that pins the location of the accident remains. The driver or stakeholders in the community can send the location of the accident too. (2) Driver assistants can be modified to use AI in wildlife conservation where an agent can detect a hotspot based on existing factors such as forests underpasses. In addition, the agent may predict the collision of animals and cars in a particular region based on factors such as weather patterns, among others. (3) Improvement of mobile mapping system 2 and the development of new solutions that focus on detecting small animals will minimize WVCs.
- Incorporating connectivity in wildlife reserves with ADSs. Connectivity in wildlife is composed of habitat connectivity which looks at the degree of movement of organisms or ecology processes, i.e., the more the movement, the more connectivity, and the less movement, the less connectivity. Landscape connectivity looks at how the landscape facilitates species movement and other ecological flows. It is important to deploy the ADS (moveable structure) at a location with high connectivity. These systems will improve detecting animals since the exact location of system deployment experiences high animal movement patterns. Such considerations will drastically contribute to eliminating roadkill. We note that a swift change in the location of moveable structures to new locations is possible should connectivity change due to different animal behaviors and change in habitats.
- Sensitizing drivers about ADSs. Training drivers about road usage and also the presence and usage of the ADS in hotspot areas is vital. This will result in reducing unnecessary WVCs, which are caused majorly by the ignorance of drivers about the ADSs on the roads.
- Open data: We recommend that conservation agencies collecting data from these existing wildlife monitoring tools loosen their rules for accessing this data for research purposes. Additionally, emphasis must focus on having a fair and balanced open dataset in wildlife conservation to ease the development of cutting-edge research in mitigating WVCs.
- Developing safe, reliable, and robust ADSs that use machine learning. The designs must be fair by ensuring bias is minimized through algorithm audits [141] and other AI ethics practical implementations. Moreover, the fairly designed algorithms should reduce privacy and security attacks, e.g., model extraction attack, model inversion attack, poisoning attack, and adversarial attack on an AI system [142]. Globally, researchers emphasize technology to be made more human-centric. For AI to be fair, work on explainability of AI [143,144] is on the rise to minimize challenges raised by centralized machine learning architectures such as privacy concerns and failure to explain and interpret the AI models. Additionally, scrutiny of AI algorithms is escalating due to the mistrust of AI, leading to the high demand for auditing frameworks [141]. Attempts made help minimize road accidents by applying AI fairness to the health sector [145]. Moreover, documented guidelines on integrating AI ethics in wildlife conservation AI systems help researchers consider AI ethics in wildlife conservation [146]. When embraced in wildlife conservation, all these attempts mitigate WVCs leading to safe, reliable, and robust ADSs.
- Venture into developing or upgrading existing ADSs and machine learning methods used in animal detection. Combine these to detect animals and mitigate WVCs. The ADSs that detect animals are geophones, UAVs, GPS, and VHT tags. Integrating these with deep CNN and other machine learning methods used so far in the detection of wildlife is vital and helps mitigate WVCs.
- Although progress is made in understanding the factors contributing to WVCs, there is still a paucity of data and information associated with not only factors but also sub-factors leading to the occurrence of WVCs. We propose future research to exhaust both positive and negative factors and sub-factors contributing to WVCs, hence generating enough literature for the secondary studies. Factors reviewed are climate change and season, animal behavior, human behavior, and road features. Proposed sub-factors under road features are road-type, width, and curve. For animal behaviors, we propose innate behaviors and learned behaviors. Sub-factors for climate change, season, and human behaviors are vital when exploring how these factors and sub-factors lead to WVCs. The factors that lead to WVCs have commonly been non-exhaustively studied in isolation. However, a more integrated approach in primary studies is needed to examine the factors leading to WVCs.
- State-of-the-art point cloud datasets together with machine learning are achieving acceptable results and are good in detecting, classifying, and segmenting objects on the road. However, these datasets are not used comprehensively in mitigating WVCs. This gap needs to merge since a huge data collection of point clouds data samples by the LiDAR sensors act as the eye of self-driving vehicles providing a 360-degree view of their surroundings to enable safe driving and hence less WVCs.
- Detecting WVC hotspots helps to inform planning to improve driver and wildlife safety. WVC hotspots can be detected using machine learning. Whenever a driver or any other stakeholder is involved in an accident or is in contact with an accident, they can take a picture and save the details on a server using a mobile application. The system’s interface should capture the image and location of the accident. Moreover, WVC hotspots detection may be by collecting road features image and video data, training computer-vision machine learning models, and deploying such models on the car dashboard. Whenever a driver is in the car and arrives at the hotspot area, the system should be able to alert the driver to reduce the speed.

## 8. RQN6: How Applicable and Feasible Are the Proposed Solutions?

**Figure 8:**
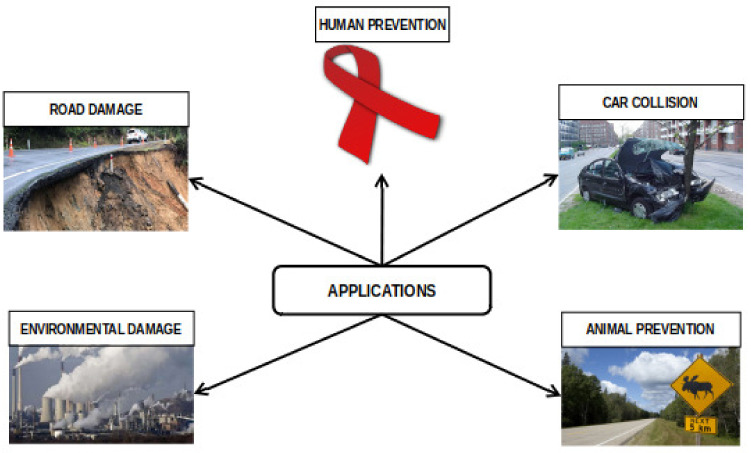
Summary to justify the applicability of the proposed solutions.

The proposed solutions are applicable because they come as a solution to minimize the issues and challenges arising from the review, hence mitigating WVCs. These solutions are applicable because of the following advantages they come with as illustrated in Figure 8:

- Road damage: On implementing the proposed solutions, we attain good results such as (1) minimizing road damages caused by rampant WVCs between animals and cars in hotspot areas, (2) minimizing costs that are associated with the road users such as transport fares charged to passengers as well as the rate at which the car components need replacement, (3) improvement on the market for farmer’s products by limiting road damage, which increases mobility and eases farmers in accessing markets for their products, (4) eases the transportation of imports and exports which later improve the country’s gross domestic product, and (5) the citizens will start experiencing less road traffic in hotspot areas.
- Accident car collision: The proposed solutions minimize not only the escalating number of WVCs but also the damage they impart on properties. Properties such as cars, items within the cars, and properties usually near the collision sites such as bridges, signposts, electricity poles, and much more wear out easily, and others become diminished due to the damage caused by WVCs. In the long run, implementing these proposed solutions leads to low costs as fewer properties are damaged, lost, and the frequency of animal carcasses minimized.
- Human prevention: The innovations proposed prevent lives of all the different stakeholders. Usually attained by minimizing death that frequently occurs due to collisions in hotspot areas. Stakeholders are such as insurance companies, highway agencies, citizens on social media, and any other persons that use road transport.
- Animal prevention: Preventing animals from road damage and death is vital. The proposed solutions ensure drivers are aware of the existence of animals on the road, which later limits collisions and minimizes the mortality rates of these animals.
- Environmental damage: The proposed solutions will minimize pollution caused by WVCs. The car collision parts and fuel smoke from cars pollute the environment. When minimizing pollution, climate change, and global warming, reduction in the extinction of animal species is limited.

**Figure 9:**
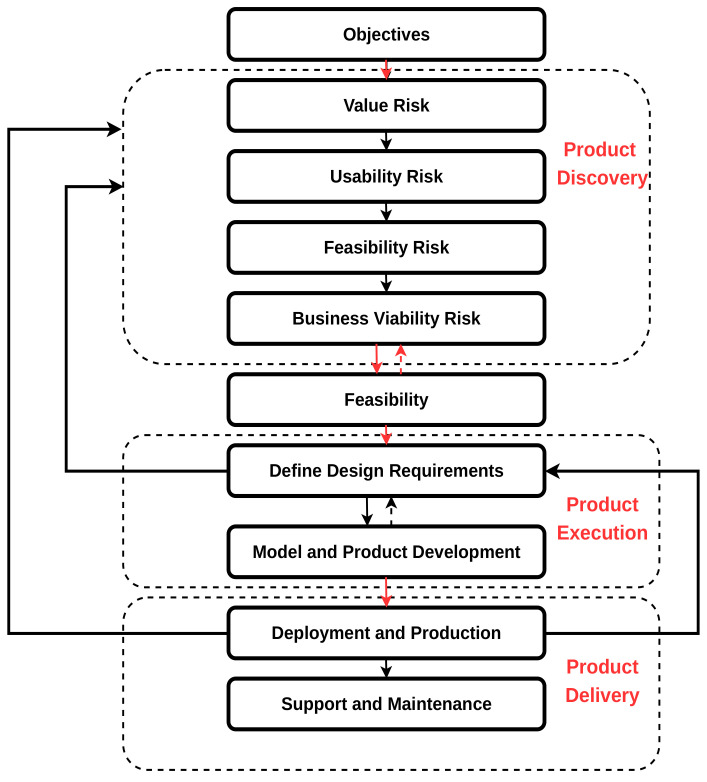
The product development lifecycle for determining feasibility analysis of machine learning projects (adopted from Cagan [147]).

The proposed systems in Section 7.2 contain machine learning functionalities. The functionalities aid in minimizing WVCs. In determining the practicability and feasibility of the proposed solutions, we propose and discuss a continuous general product development lifecycle in Figure 9. The workflow proposes steps to follow to determine the feasibility of machine learning systems, i.e., whether the project defined is feasible or not. To conduct a feasibility analysis for any machine learning project, product managers should follow the proposed workflow from product discovery, feasibility, product execution to product delivery. This workflow enables project managers to guide team members during the continuous process of product discovery to product delivery. In Figure 9, we describe the product development lifecycle steps to follow when developing machine learning models and products. It has three continuous phases, product discovery, product execution, and product delivery, which are vital [147].

- Product Discovery: The discovery phase helps product managers to assess good ideas to consider for the final project. The output of the product discovery is the validated product backlog. The validated product backlog states four risks that every product needs to consider and address. Risks are tackled upfront rather than at the end. In modern teams, the risks are tackled before deciding on building anything. These risks include: (1) value risk, whether customers will buy the product or users will choose to use the product, (2) usability risk, whether users can figure out how to use the product, (3) feasibility risk, whether our engineers can build what we need with the time, skills, and technology we have, and (4) business viability risk, whether the solution also works for the various aspects of our business.
- Feasibility: The key objective is to evaluate all key factors relevant to a defined project after tackling the significant four risks. The components may be technical and non-technical, but all of these aid in predicting the likelihood of project success. When the non-technical factors are acceptable in the feasibility analysis, technical aspects are further processed and are modeled to quickly obtain a baseline model performance for the task defined while showing measurable progress on the selected baseline. The baseline model developed may rapidly use hyperparameter tuning to obtain different model architectures using an iterative process. If engineers work on a novel solution, the feasibility is more complex than upgrading and updating an existing system. Finally, the results from the feasibility analysis inform the decision on whether to proceed to the next step in the product lifecycle, i.e., product execution.
- Product Execution: Whenever the developed feasibility prototypes show that the performance would likely be acceptable, product managers engage the development team to execute the idea according to client needs. Products are defined and designed collaboratively rather than sequentially. In executing the project, the project team may follow the steps: (1) Define Design Requirements: After the feasibility analysis, project managers define the requirements of the system. A requirement is a need, functionality, or characteristic of a system [148]. A functional specification document describes the requirements to be implemented by the software solution. The document captures what the software needs to perform to support a user. The design requirements help product development teams prioritize work and decide what to build next. Following are two main types of requirements: (a) Functional requirements are those that must be met to deliver the project and are described from the customer’s point of view, e.g., how will the customer experience it and/or benefit from it? Specific functional requirements may define each application in the machine learning domain. Some machine learning applications are credit card detection, whose functional requirement is a count of false positives and classification. The functional requirement for classification is a threshold on a count of low-confidence predictions that require human review and approval. (b) Non-functional/technical requirements define the system quality and determine the implementation of the system. Non-functional/technical requirements are system qualities such as security, accuracy, performance, security, data privacy, costs, availability, maintainability, operability, and scalability. (2) Model and Product Development: After defining design requirements, these, together with the selected aggregated data, act as input to the model and product development phase to build a baseline model or product. Later, evaluations of model output determine if the performance is acceptable. If yes, the model and product are deployed and sent to production. If no, a loop is activated to repeat the process from product discovery to product delivery.
- Product Delivery: The process is continuous and enables delivery of executed products to the market, meeting the necessary performance, reliability, and fault. This phase focuses on implementing a solution that solves an underlying problem. They typically focus on attaining the expected results.
- Production and Deployment: When the experiments are satisfactory, the model is deployed and set to production for clients to use.
- Support and Maintenance: This is the last step after model deployment and production. It can be challenging when conducting support and maintenance of machine learning products over time because machine learning is tightly coupled and integrated, meaning it depends on each of the components in the framework. Changes in one component, feature space, hyperparameters, and learning rate affect the model’s performance. We need maintenance to track these issues and rectify them before they go astray.

Even though we find the proposed solutions novel, feasible, and also practical, they still need more case-by-case justification of the feasibility of each proposed solution. We, therefore, recommend future studies to verify the proposed systems’ practicability and feasibility simply because feasibility analysis of a system is different for each project and location. It is essential to verify feasibility across different continents, countries, domains, weather conditions, types of animals, and road types. While verifying, the project teams may develop system prototypes and/or implement product development lifecycles for software and embedded systems.

In summary, Section 7.1 discusses issues and challenges arising from the review, Section 7.2 proposes future research directions and a way forward, and Section 8 explains the applicability of the proposed solutions.

## 9. Conclusions

The emphasis on avoiding WVCs is a big concern in conservation. In mitigating WVCs, traditional methods such as underpasses, overpasses, plus fencing have been implemented and have contributed significantly to the decline of WVC mortality rates. Even when these traditional methods have recorded positive results in mitigating WVCs, they still have extreme limitations. These limitations have prompted the rise of ADSs such as area cover, mobile mapping, break-the-beam, and buried cable systems. These systems can even send an alert in case of intrusion in the detection area to caution the driver. However, these ADSs have also registered some limitations such as (1) failure to detect small animals, (2) false detection of animal species, and (3) ignorance of drivers on-road usage. A few works have introduced and implemented machine learning algorithms and datasets to help and overcome these limitations.

This paper aimed at generating rules that analyze how different factors contribute to WVCs and how the rules integrate machine learning to prevent WVCs. Previous solutions such as ADSs have contributed significantly to conservation research. In this review, we have criticized research on factors that influence WVCs, machine learning algorithms, ADSs, and datasets currently implemented to prevent WVCs. We have highlighted the issues and challenges arising from the review and proposed future solutions. We also noted that most of the current systems implemented to mitigate WVCs do not detect small animals. We suggest developing solutions in Section 7.2 to improve in detecting animals with a core goal of mitigating WVCs and further justify and determine the feasibility of the projects using the proposed continuous product development lifecycle.

Finally, we agree that animal detection and animal warning systems have contributed a positive impact on reducing WVCs. However, more research is needed to address the wildlife conditions in Africa. The automated systems should be developed and tested in Africa since Africa has different animal species and weather patterns than the Northern Hemisphere.

## Figures and Tables

**Table 1:** Research questions and their descriptions. RQN*i* represents the research question number *i*.

RQN*i*	Question	Description
RQN1	What are the negative factors that lead to the occurrence of WVCs?	To identify negative factors that contribute to human–wildlife conflicts and WVCs.
RQN2	What ADSs are deployed to mitigate WVCs in the primary studies?	To identify all the ADSs and techniques used in the selected primary studies. State the results, strengths, and weaknesses of each system.
RQN3	What types of datasets are currently used to mitigate WVCs?	The different types of datasets, e.g., text, images, time series, etc., and what machine learning tasks can use the data identified.
RQN4	What types of machine learning algorithms are used to mitigate WVCs?	To identify all the machine learning algorithms used in the selected primary studies to mitigate WVCs.
RQN5	What are the limitations of the primary studies in mitigating WVCs?	To identify all the constraints or weaknesses of the ADSs and machine learning methods in mitigating WVCs and propose future recommendations.
RQN6	How applicable and feasible are the proposed solutions?	To show how the solutions minimize the issues and challenges arising from the review.

**Table 2:** Search terms used when retrieving manuscripts from the different internet sources.

General Term	Term
break-the-beam + animal-detection systems	break-the-beam/animal-detection systems/mitigation/roadkill/animal-vehicle collisions and wildlife-vehicle collisions
area-cover + animal-detection systems	area-cover/animal-detection systems/roadkill/mitigation and/animal-vehicle collisions and wildlife-vehicle collisions
mobile-mapping + animal-detection systems	mobile-mapping/roadkill/animal-detection systems/mitigation/animal-vehicle collisions and wildlife-vehicle collisions
buried cable + animal-detection systems	buried cable/roadkill/animal-detection systems/mitigation/animal-vehicle collisions and wildlife-vehicle collisions
driver assistance + animal-detection systems	driver assistance/roadkill/animal-detection systems/mitigation/animal-vehicle collisions and wildlife-vehicle collisions
unmanned aerial vehicles (UAVs) + animal-detection systems	unmanned aerial vehicles (UAVs)/animal-detection systems/roadkill/mitigation/animal-vehicle collisions and wildlife-vehicle collisions
drone + animal-detection systems	drone/animal-detection systems/roadkill/mitigation/animal-vehicle collisions and wildlife-vehicle collisions
machine learning methods + animal-detection systems	machine learning methods/animal-detection systems/mitigation/roadkill/animal-vehicle collisions and wildlife-vehicle collisions
datasets + wildlife-vehicle collisions	datasets/wildlife-vehicle collisions/animal-detection systems/mitigation/roadkill/animal-vehicle collisions and wildlife-vehicle collisions

**Table 3:** Strengths and limitations of cognitive theories: TRA and TPB models.

Model	Strengths	Limitations
TRA	Subjective norms and attitudes toward predicting behavior were important Future behaviors were determined by behavioral intentions	The theory focuses on behavior under volitional control The model is not used due to the theory of planned behavior
TPB	The introduction of perceived behavioral control covers the non-volitional behaviors that were eliminated in the theory of reasoned action Gaston et al. [82] highlighted the theory as good at explaining intentions	Intention prediction is more than behavior prediction. This is due to temporal element [75]

**Table 4:** Description of sensors deployed in area cover systems.

Sensor Type	Description
Active infrared sensors [106]	Provides its own source of energy for illumination It has a transmitter which emits invisible active infrared beams and the receiver which receives and analyzes the beams Detects intruders passing between the detection area
Passive infrared sensors [107]	This sensor works by detecting the energy emitted by other objects such as an animal This sensor uses the concept of black body radiation The objects emit heat in the form of infrared radiation in the detection area Any intruders in the detection area are detected
Passive video sensors [106]	This technology detects and records objects and evaluates scenes recorded by a video camera
Active microwave radio sensors [106]	This technology uses both passive and/or active remote sensing It emits microwave pulses and measures the reflection of a moving object They cover a larger area than infrared sensors Are vulnerable to electrical interference

**Table 5:** A comparison of areas cover systems in terms of their sensor types, performances, strengths, and weakness.

Reference	Year	Sensor/Signal Type	Results	Strengths	Weakness
Desholm [24]	2003	Thermal infrared	TADS can record migrating birds approaching the rotating blades of a turbine, even under conditions with poor visibility	The study achieves good visibility with thermal cameras during severe fog and snowfall Good quality of compressed and non-compressed captured images A threshold temperature level captures only sequences of birds Vibrations from turbines did not affect the video recording Time delays 20 s during remote controlling had no practical problem	We recognized water condensing within the metal box as a potential problem Collisions of birds and offshore wind farms are expected to continue during periods of bad visibility The study was on only eider duck, so limited data of other species
Gordon et al. [25]	2004	Infrared Speed	Detects a deer Captures details about the vehicle and its speed Categorized vehicles such as automobiles and semi tractor-trailers Able to store and retrieve sensor data from the global system for mobile communications Flashing warning lights activated to alert drivers	The FLASH detects deer moving through the crossing area Minimizing of speed reductions is successful when tested during different times of the day, months of the year, date, treatment, among other factors	The system is not very effective and needed station on the road with high traffic Poor usage of the system by drivers due to lack of an educational program to train users of the system on how to use it
Huijser et al. [17]	2006	Microwave radio	Detects animals within a certain range of a sensor	Save the data for the detection to use it for decision making	Detection delay
U.S. Department of Transport [109]	2008	Passive infrared and video Active (microwave radar)	Detect large animals	Liberty to wildlife movement than traditional fencing systems	Experiences excessive false positives
Huijser et al. [18]	2009	Passive IR	Detection of animals based on heat and motion	Detect large animals Large detection areas compared to break-the-beam	Cannot detect small animals System is less sensitive during high winds False detections
Mukherjee et al. [27]	2013	Passive infrared Short-range radar	Radar-based system is placed at lower height from the ground than an infrared Radars cover longer-range monitoring than infrared cameras Radars use fewer sensors and poles to cover roadway than infrared	Able to monitor animals beyond the virtual tripwire No precise alignment of transmitter–receiver pairs Installations can be in ridges, gullies, and rocky outcrop landscapes	Infrared systems operate only at night Higher false alarms during the day Prone to false detection due to rain Possibility of an animal becoming stuck inside the fenced section or roadways Delays in detection and sending alerts Longer monitoring of detected animals (radar)
Mukherjee et al. [27]	2013	Radar	Large animal detection (LAD) reliably detects large animals in covered area Activates flashing beacons to alert drivers Influence of driver behavior so that drivers can avoid a collision	LADs deliver high reliability by reducing false alarms in detecting animals Achieves a reliable and accurate tracking in a low power Cost-effective and environmentally friendly manner It is a modular system, which makes it easy to upgrade with less effort	Delays in detection
Vikhram et al. [26]	2017	Infrared Sound	Passive infrared and ultrasonic sensor detect an animal in detection area and send an alert to the controller presence of the animal and send an input signal to the controller Audio voice recorder and playback plays the sound to divert the animal At night when a flashlight is on, an alert to the forest department and farmer about the presence of an animal is made Solar powers the system	The design system will not be dangerous to animal and human being, and it protects farm	The system is not extensively tested, under bad weather (rain, snow, etc.)
Huijser et al. [23]	2017	Doppler radar Thermal camera Sensor	Detects large animals (in 113 m) Radar detects a person, and the warning signs are on as long as the person was still in the detection area	The system can further be programmed to detect large animals in a much longer distance (400 m) The high location of the Doppler radar reduced the risk of theft, vandalism, or accidental damage and certain types of false alarms Warning signs are activated whenever the system detects an animal Drivers informed about the detection zone a distance away from the detection area Detection of one animal multiple times while it is still present in the detection area This technology distinguishes between large mammals and other objects such as vehicles	False positives: the radar reports the detection, yet there is no large animal False negatives: the radar did not report any detection, yet there is a large animal
Shapoval et al. [22]	2018	Doppler radar HB100	Radar is able to detect dog and human when placed behind the crops	System is able to detect these animals under different gardener crops placed in front	Generally, the system did not perform well in detecting objects under thick crop cover and it achieves unfavorable results on *p*-value

**Table 6:** A comparison of break-the-beam systems in terms of their sensor types, performances, strengths, and weakness.

Reference	Year	Sensor/Signal Type	Results	Strengths	Weakness
Huijser et al. [17]	2006	Microwave radio	Detection of an animal when it breaks the beam of light Activated warning signs to alert driver of existence of an animal on the road Increase awareness and reduction in speed of vehicle, hence less collisions	Increase in the alertness of drivers which leads to fewer or less severe WVCs	Curves, slopes, and vegetation landscapes require additional sensors which increases the cost of the system
Huijser et al. [18]	2009	Infrared Laser Microwave radio	Four systems in test-bed are able to detect large animals	Can detect large animals Excellent visibility, hence low number of false positives	Cannot detect small animals False positives due to sensor moving in and out of the alignment Low visibility may block or reduce the narrow signal path of optical break-the-beam systems
Huijser [20]	2010	Microwave	Detection of animals are on a higher percentage Records date and time of each individual detection Records animal movement within the enclosure	Less error rate with 1 false positive and 4 false negatives out of 140 valid detections The system achieves an overall accuracy of 97%	Challenge in detecting small animals
Grace et al. [21]	2016	Infrared-beam	System performs from poor to fair in detecting target animals Flashing light triggers when the beam breaks, this alerts drivers of the presence of an animal on the highway	Reduction in the number of false positives by nearly 50% through the use of a control site to test the RADS	Large number of false positives Encountered system malfunctions that affected the results of the system Ignorance of the system by the drivers, public education, increase in public understanding, and driver awareness is needed to achieve better results
William et al. [19]	2019	Microwave sensors	KNN accuracy greater than rain forest	Method is able to identify animals	Small sample of dataset System is not tested on real-life images but on synthetic images System was not deployed to conduct more test

**Table 7:** Summary of results, strengths, and weaknesses of BCADS, roadkill detection, driver assistance, autonomous vehicles, and mobile mapping ADSs.

Reference	Year	Application	Results	Strengths	Weakness
Huijser et al. [28]	2012	BCADS	All intrusions in the detection area are detected with a high percentage of 98.12%	The system responds quite well when tested under different environment conditions, it generated no false positives and very few false negatives False positives are 0% on testing reliability of the system	Reliability of the systems results are still not favorable on the number of false negatives (0.46%)
Druta et al. [29]	2015	BCADS	System detects large- and medium-sized animals crossing the road Provides data on the location of animal detected along the length of the cable	Detects animals with 95% accuracy during nighttime and under all-weather conditions	System still registers false detections In some cases, when a vehicle crosses the edge line and was entering the detection field, an alarm triggers and is declared as a regular intruder detection. To some extent, the signal response depends on the vehicle distance from the detection field Nuisance alarm triggers are due to water movement on the cable path
Gil et al. [35]	2016	Road-kill detection	Scan images of amphibians on-road and GPS coordinates Capture amphibians and road surface images Data storage and data processing All these devices are synchronized using an encoder attached to the left rear wheel of the trailer	Mobility Versatility in being easy to attach to any vehicle Motion stability needed for the image capturing system and thus the dual axle system Standalone powering over several hours of utilization Accommodation of all the equipment with protection to rain and dust Capability to scan the road surface with high resolution of a complete track (∼25 km) at an acceptable speed	Need to increase the accuracy of the system, on using the Haar classifier method of computer vision which achieves 65% accuracy which was low
Sharma and Shah [31]	2017	Driver assistance	The algorithm can detect an image, video, and the distance between the animal and car. Send alerts to the driver distance away before the animal and car move closer to each other, hence minimizing collisions	The algorithm can detect animals in both a video and image	The study uses limited dataset, hence need for improved performance Not tested object detection during the night
Rosenband [32]	2017	Autonomous vehicle	Delivers autonomous self-driving without requiring humans	These cars are able to detect objects on the road by adapting to different environment	The cars have registered accidents with humans, animals, etc., and also maneuvers [111]
Sillero et al. [33]	2018	A road mobile mapping device for supervised classification	The algorithm is able to detect amphibians from the amphibian dataset in all the three cases	The classification algorithm can adopt to any classification group The system can work day and night It is ideal for passive surveys	The work still faces false-positive detection
Guedes et al. [34]	2019	Mobile mapping system 2	System detects approximately 78% amphibians and birds (overlooking 22%)	The survey can be conducted by any person using mobile mapping system 2 with or without sampling skills	Generated 17% of false positives
Goswami, et al. [110]	2019	Driver assistance	Three algorithms (Mask R-CNN, YOLOv3, and MobileNet-SSD) are able to detect animals on the road	MobileNet-SSD performed better in detecting animals	The study uses a small dataset The algorithm is not deployed on the driver assistance and tested
Druta et al. [30]	2020	BCADS	The system identifies crossings of large- and medium-sized animals and provides data on their location along the length of the sensing cable	BCADS prove to be effective and reliable when evaluating under controlled and secure conditions Sometimes the system is able to detect small animals Able to detect small, medium-sized, and large animals with approximately 99% reliability	Total 20% of the drivers do not respond to the warning signs, which is dangerous, as this can lead to WVCs False negatives—this is when a target crosses over the cable or is inside the detection zone and an alarm is not triggered

**Table 8:** Summary of datasets that are used in mitigating WVCs showing types of dataset, task (✓ means task is performed on data and × means a task we have proposed that can be performed on data and no work in the literature has performed the task), collectors, collection period, number of instances, and country (− means authors did not state data collection period, instances, and country).

Dataset	Type of Data	Task	Collectors	Collection Period	#Instances	Country	Ref Works
Animal-related crash data [113]	Time series	Prediction × Forecasting × Classification ×	Experts	2001 to 2007	0 to 4285 per year	Australia	None
Cheetah, Hyena, Jaguar and Tiger dataset (https://www.kaggle.com/c/swdl2020/data, accessed on 1 September 2021)	Images	Object detection × Classification × Segmentation × Localization ×	Experts	2020	1000	−	None
Data on roadkill [114]	Time series	Prediction × Forecasting × Classification ×	Experts	2007 to 2018	1436	Kenya	None
Kangaroo dataset (https://www.kaggle.com/hugozanini1/kangaroodataset, accessed on 14 August 2021)	Images	Object detection ✓ Classification × Segmentation × Localization ×	Kaggle	2020	313	Scraping online	Yi [41]
Oregon wildlife dataset [119]	Images	Object detection ✓ Classification × Segmentation × Localization ×	Kaggle	2019	2029	Oregon	Fan et al. [120]
Pothole image dataset [117]	Images	Object detection ✓ Classification × Segmentation × Localization ×	Kaggle	2019	618	Scraping online	Fan et al. [120]
Roadkill data [115]	Time series	Prediction × Forecasting × Classification ×	Experts	1990 to 1991	183	Tanzania	None
Roadkill dataset [116]	Time series	Prediction × Forecasting × Classification ×	Citizen science	2011 to 2014	2642	South Africa	None
Serengeti dataset (https://lila.science/datasets/snapshot-serengeti, accessed on 20 September 2021)	Images	Object detection ✓ Classification × Segmentation × Localization ×	Experts	2010	7.1M	Tanzania	Marco et al. [121], Sadegh et al. [122]
Snake roadkill [39]	Time series	Prediction ✓ Forecasting × Classification ×	Citizen science	2006 to 2017	>40,000	Taiwan	Yue et al. [39]
Stanford cars dataset [118]	Images	Object detection ✓ Classification × Segmentation × Localization ×	Kaggle	2018	16185	USA	Fan et al. [120]
Waymo open dataset [124] (http://www.waymo.com/open, accessed on 11 December 2021)	Images (LiDAR and camera)	Detection and tracking ✓	Experts	−	1150	USA	Sun et al. [124]
Wildlife-train collisions data [125]	Sounds	Train detection ✓	Experts	−	183	Canada	Backs et al. [125]
WVC hotspots [112]	Time series	Prediction ✓ Forecasting × Classification ×	Citizen science	2014 to 2016	529	Italy	Valerio et al. [112]
YOLOv3 dataset (https://pjreddie.com/darknet/yolo/, accessed on 9 December 2021)	Images	Object detection × Classification × Segmentation × Localization ✓	Experts	2018	−	USA	Redmon et al. [123]

**Table 9:** Compared results of correctly classified animals from an animal dataset with 5 classes (Results from Tibor et al. [38]).

Accuracy of Correctly Identified Animals for Each Class (%)					
	Bear	Wolf	Fox	Deer	Hog
PCA	82	79	78	76	82
LDA	81	77	78	81	83
LBPH	85	87	83	84	82
SVM	87	864	85	83	81
Proposed CNN	97	95	95	93	91

**Table 10:** Comparison of machine learning methods used to mitigate WVCs.

Reference	Year	Method	Data	Application	Results	Strengths	Weakness
Parikh et al. [127]	2013	Feature extraction Frame differencing Template matching algorithm	Videos of birds Target images Template images	Real-time application	A 94% accuracy in detecting birds A 5.94 % false positive rate for bird detection A 5.23% false negative rate for bird detection	Bird detection with high accuracy This real-time system can be adapted to detect large animals before they enter the road to help mitigate WVCs May help to save crops in farm from animals, hence mitigating human–wildlife conflicts	System still experiences false detections
Tibor et al. [38]	2017	CNN PCA LBPH SVM LDA	Images of wolf, bear, deer, fox, and hog	Animal recognition system	Highest accuracy scored models: 97% on bear by CNN, 82% on bear and hog by PCA, 83% on hog by LDA, 87% on wolf by LBPH, and 87% on bear by SVM	Highly predicted the class labels	No domain applicability stated Failed to classify some animals
Antonio et al. [19]	2019	KNN Random forest	Synthetic images	Simple ADS	KNN accuracy is greater than random forest	Method is able to identify animals on the road	Dataset was too small Images used are not real-life The system is not deployed and tested on roads The accuracy of the system was slightly above average
Guedes et al. [34]	2019	CNN VGG16 VGG19 ResNet50 Inception Xception	Images of amphibians and birds	Mobile mapping system 2	Detects 78% amphibians Overlooked 22% birds A total 17% false positives The algorithm classified 37.1% of the detected animals to the species level (the remainder was classified as “others”)	Mobile mapping system 2 better than previous system mobile mapping system 1 High detectability Detects small animals	It still requires human intervention to determine if the animal is alive or road-killed The weather conditions may affect the images’ quality The system is not suitable to use at night: there is no light system as in the previous version A high storage capacity is necessary (the tests originated 1.65 terabytes of images) Specialized people needed for image processing and animal identification A workstation is required for computing heavy data
Yue et al. [39]	2019	Maximum entropy environmental niche modeling	Roadkill snakes images	Snake roadkill mitigation	Detected road-killed snakes	They sighted patterns across snake species differing in habitat use, foraging behavior, and taxonomic group	Natural history and landscape factors contribute a lot to the increase in number of snake roadkill
Banupriya et al. [40]	2020	CNN	Wildlife images, e.g., elephants and cheetahs	Animal detection	The model detects a cheetah at 79% accuracy and an elephant at 86%	Able to classify animals correctly with a good accuracy	No warning to the driver about the detected animal Algorithm was not tested on roads
Yi [41]	2020	YOLOv3	Kangaroo dataset (https://www.kaggle.com/hugozanini1/kangaroodataset, accessed on 14 August 2021) YOLOv3	ROOD System	Developed a roadkill alert system that mitigates roadkill and alert drivers of high risk areas	Data collected help policy makers, road authority, and biologists to respond to collisions	This system is not installed on real cars

**Table 11:** Summary of issues, challenges, and future research directions.

Current Issues and Challenges from the Review	Current systems are not suitable for small and medium-sized animals Machine learning algorithms do not detect individual species and their behaviors but generalizes species High false detection of existing systems Some systems fail to alert drivers about the presence of animals on the road Failure to detect hotspot areas Investigations on ethics in AI in wildlife conservation is not explored Current ADSs are expensive to implement ADSs do not exist in South Africa and Africa Limited data sets for training AI models MMS needs human intervention to detect small dead animals Landscapes with curves, e.t.c. need more hardware tools Driver ignorance on the usage of roads and ADSs
Proposed Future Research Directions	Design matching algorithms that detect species vulnerable in specific season and month of the year An update and upgrade of the existing system is essential Improved vision algorithms to detect each species Development of other new and improved efficient solutions Incorporating connectivity in wildlife reserves with ADSs as a consideration Sensitizing drivers about ADSs Enable open data for research in wildlife conservation Develop safe, reliable, and robust ADSs that is fair and accountable Venture into other machine learning methods and ADSs, e.g., geophones, UAVs, GPS, and VHT tags. Integrate them to work towards mitigating WVC Development of real-time ADS Conduct exhaustive primary studies on factors and sub-factors contributing to WVC Use 3D point cloud data sets in mitigating WVC Detecting WVC hotspots

## Data Availability

Not applicable.
